# Germination-Related Universal Stress Protein (GRUSP) Is Involved in Abscisic Acid Responses During Imbibition and Early Post-Germination Growth of *Arabidopsis* Seeds

**DOI:** 10.3390/ijms27177540

**Published:** 2026-08-23

**Authors:** Elena S. Pojidaeva, Darya S. Gorshkova, Natalia V. Kudryakova, Victor V. Kusnetsov

**Affiliations:** K.A. Timiryazev Institute of Plant Physiology RAS, 35 Botanicheskaya St., Moscow 127276, Russianvkudryakova@mail.ru (N.V.K.); vkusnetsov2001@mail.ru (V.V.K.)

**Keywords:** Arabidopsis, abscisic acid, At3g58450, germination, GRUSP

## Abstract

The universal stress protein (USP) family is a large but poorly studied group of plant proteins whose roles are considered primarily in the context of stress tolerance. This study demonstrates that the functions of USPs are not limited to the stress response but also influence plant developmental processes, namely, the regulation of seed germination and seedling establishment. Using reverse genetics, we found that disruption of the *Arabidopsis thaliana At3g58450* gene, encoding germination-related USP (GRUSP), results in delayed germination and abscisic acid (ABA) hypersensitivity in the mutant the GABI-kat 115C08 (*grusp-115*) knockout line. This hypersensitivity was only partially rescued by the application of fluridone, an ABA biosynthesis inhibitor. Altered ABA content and expression patterns of genes involved in ABA metabolism and signaling, along with a decrease in the mRNA levels of gibberellin (GA) oxidases in dry and either Murashige and Skoog (MS)- or ABA-imbibed mutant seeds, indicate that GRUSP function is primarily associated with seed imbibition and early germination stages. Furthermore, the *grusp-115* mutant showed upregulation of *ABI5* transcripts in imbibed seeds and seedlings as well as an accumulation of ABI5 protein level under excess GA conditions, indicating a functional relationship between these proteins. Hence, GRUSP is a novel component of the GA and ABA pathways whose function is initiated during seed germination.

## 1. Introduction

Seed dormancy and germination are tightly regulated by both internal and external cues. The balance between gibberellins (GAs) and abscisic acid (ABA) is the main endogenous factor that regulates the physiological state of the seed (desiccation tolerance, dormancy, germination, and longevity) [1]. GAs stimulate germination, whereas ABA maintains seed dormancy [2]. Antagonistic interactions between ABA and GAs occur through the regulation of phytohormone metabolism and signaling pathways [3]. GA metabolism is regulated mainly at the transcriptional level of 2-oxoglutarate-dependent GA dioxygenases. These enzymes, namely, GA20 dioxygenases (GA20ox) and GA3 dioxygenases (GA3ox), catalyze the final biosynthetic steps [4,5], while GA2 dioxygenases (GA2ox) mediate GA deactivation [6]. Similarly, the expression of key ABA biosynthetic enzymes, such as zeaxanthin epoxidase (ZEP) and the 9-cis-epoxycarotenoid dioxygenase (NCED) family, positively regulates ABA metabolism [7,8], whereas ABA catabolism is predominantly driven by ABA 8′-hydroxylases (cytochrome P450 monooxygenases) encoded by the *CYP707A* gene family [9]. Therefore, the dormant state corresponds to a decrease in the levels of *CYP707A*, *GA20ox*, and *GA3ox* transcripts, as well as an increase in the expression levels of the *ZEP*, *NCED*, and *GA2ox* genes (reviewed in Liu et al., 2018 [3]). This pattern of gene expression is reversed in germinating seeds and young seedlings, mediated by specific regulatory proteins [10]. For example, the ABA-dependent APETALA2-like transcription factor CHOTTO reduces GA content through the repression of *GA3ox* genes [11]; on the other hand, the GA-related RING-H2 factor XERICO stimulates ABA biosynthesis [12,13].

The GA/ABA ratio is strongly dependent on their respective signaling pathways. The primary principle of GA signaling is that GAs induce the degradation of DELLA proteins, which act as negative regulators of GA responses [14,15]. Conversely, ABA perception triggers the activation of SnRK2 kinases, leading to the induction of ABA-dependent transcription factors, mainly ABI3, ABI4, and ABI5 [16,17,18]. In general, DELLA stability and ABI3/4/5 activity represent critical nodes in the interaction between ABA and GA. Accordingly, the relationship between DELLA and ABI3/4/5—and hence seed developmental status—is governed by a network of numerous regulatory factors, including still-unidentified components [19,20,21,22].

Transcriptomic analyses of seeds have identified several potential novel regulators of germination-related pathways. Members of the universal stress protein (USP) family are found among them [23,24,25,26]. USPs were first discovered in bacteria, where they are upregulated in response to growth arrest under various abiotic stresses [27]. Typically, USPs contain a specific USP domain with a nucleotide-binding, Rossmann-like fold (adenine nucleotide α-hydrolase-like domain, PFAM: PF00582). In addition to nucleotide binding and hydrolysis, USP domain-containing proteins participate in hydrophobic protein-protein interactions [28,29]. These features suggest that USPs are versatile components of several regulatory pathways, which is supported by experimental data in prokaryotes [30,31].

Bioinformatics’ analysis identified proteins containing a USP-like domain in plants [32]. Subsequent experiments, including studies on responses to salt, drought and other abiotic stresses [33,34,35,36], as well as the identification of characteristic stress-responsive promoter motifs, have confirmed the stress-related functions of USPs [37]. However, transcriptional data indicates a broader functional range for USPs beyond stress adaptation. Most characterized plant *USP* genes are regulated by phytohormones [37,38,39]. For instance, differential regulation of *USP* homologs has been observed in transcriptomic studies of (i) the gibberellin-deficient *ga1-3* mutant [25], (ii) the brassinosteroid-deficient mutant *dwf1-6* [40], and (iii) floral homeotic *apetala3*/*pistillata* mutants [41]. A notable example is the STUNTED receptor-like cytoplasmic kinase, which contains a USP-like domain; this protein acts downstream of the GA-DELLA signaling pathway and mediates cell cycle regulation by GAs [42]. Integrating these transcriptional data with the biochemical characteristics of the USP domain itself, we propose that USP family members may serve as molecular switches regulating phytohormone crosstalk.

Analysis of the *Arabidopsis thaliana* (*Arabidopsis*) genome revealed the *At3g58450* gene as a potential member of the USP family [32], encoding germination-related USP (GRUSP), also designated as USP18 [36], whose function is associated with the seed stage [43,44]. According to the transcriptomic data, *At3g58450* is induced in GA-deficient seeds of the *ga1-3* mutant [25]. Previously, we demonstrated that loss of the *GRUSP* results in a GA-deficient phenotype characterized by reduced basal mRNA levels of GAs biosynthetic genes, low sensitivity to exogenously applied GA, and higher basal accumulation of the GAI (Gibberellic-Acid Insensitive) protein, a DELLA-family repressor of the GA signaling pathway. Additionally, the mutant showed delayed flowering under a long-day light regime, decreased levels of endogenous bioactive GA_1_ and GA_3_, and upregulated expression of the floral repressor FLC (Flowering Locus C) compared to the wild type [45].

Conversely, *GRUSP* expression is downregulated in ABA-insensitive seeds of the *abi4* and *abi5* mutants [24,26] and in drought-stressed seedlings of the *areb1/areb2/abf3* triple mutant [46]. Also, inactivation of GRUSP led to ABA sensitivity of seeds [43]. Therefore, the mutation caused a pleiotropic effect, demonstrating the role of GRUSP at different stages of plant development. Here, we hypothesize that the GA-dependent pathway in the *grusp* knockout mutant is not directly disrupted but rather is altered indirectly due to an impaired ABA-mediated response.

In this context, our present study focused on the processes occurring in dry and germinating seeds to identify the initial site of action of this protein and to analyze ABA-dependent reactions in the *GRUSP*-deficient mutant. Using a reverse genetics approach, we found that disruption of *At3g58450* (*GRUSP*) resulted in delayed germination and ABA hypersensitivity of the *grusp-115* knockout seeds compared to Col-0. These effects were accompanied by decreased transcript levels of ABA catabolic and GA oxidase genes, as well as increased expression of ABA signaling genes. Consequently, we propose that GRUSP acts as a negative regulator of ABA-dependent processes, thereby contributing to seed germination and post-germination growth.

## 2. Results

### 2.1. The Expression of GRUSP Is Downregulated upon Imbibition and During the Early Stages of Germination with the Participation of Phytohormones

**Figure 1:**
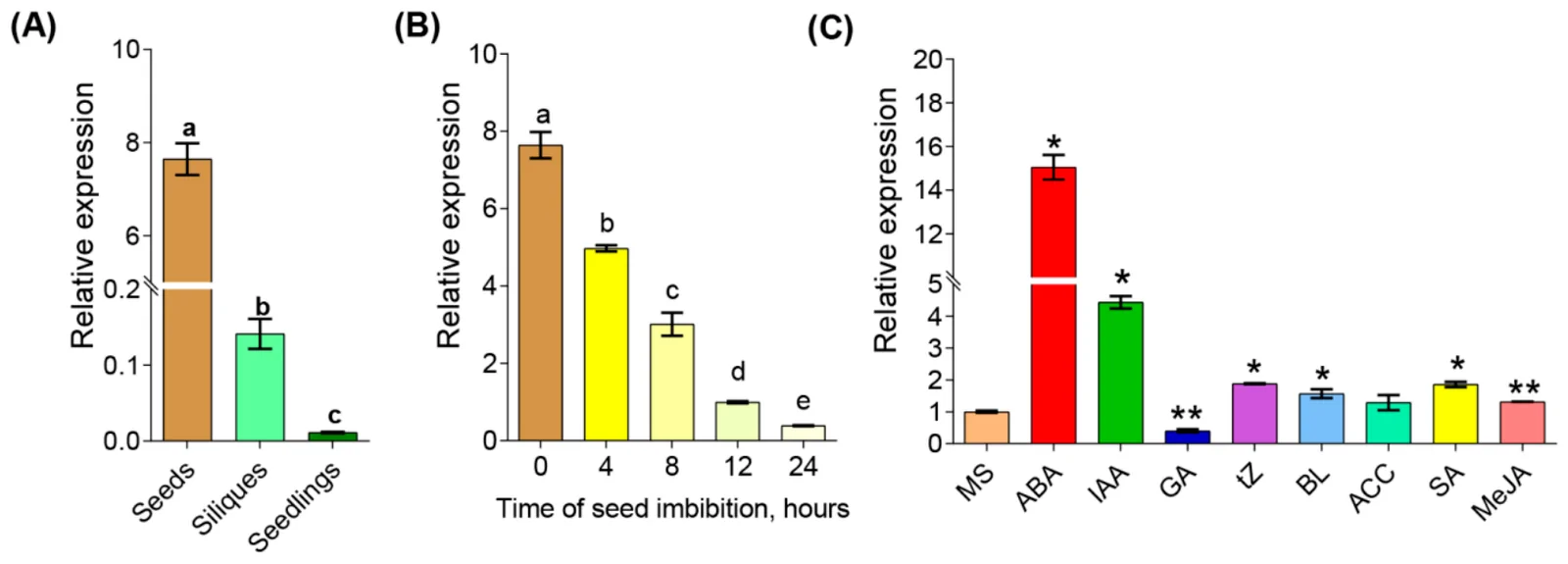
Relative expression profile of the *GRUSP* gene in Col-0 *Arabidopsis* plants. (**A**) Changes in the *GRUSP* expression level at different developmental stages. qRT-PCR analysis was performed with *CRUSP* cDNA templates prepared from dry seeds, 7-day-old seedlings and green siliques. Relative units were normalized to the reference gene *PPA2* (for siliques and seedlings) and *Ubq* (for dry seeds). (**B**) Downregulation of the *GRUSP* mRNA level upon imbibition. The seeds were imbibed in water for a 24 h time course (4, 8, 12, and 24 h) under a 16 h light/8 h dark photoperiod at 23 °C with 100 μmol photon m^−2^ s^−1^. Zero point (0) corresponds to dry seeds. Relative units were normalized to the reference gene *UBC10*. (**C**) Effects of phytohormone treatments on the expression level of *GRUSP*. qRT-PCR was performed with RNA samples extracted from 7-day-old seedlings exposed for 3 h in a growth chamber to 5 × 10^−5^ M ABA, 10^−6^ M indole-3-acetic acid (IAA), 5 × 10^−6^ M GA_4+7_, 5 × 10^−6^ M trans-zeatin (tZ), 10^−7^ M brassinolide (BL), 10^−5^ M 1-aminocyclopropane-1-carboxylic acid (ACC), 1 × 10^−5^ M salicylic acid (SA), or 5 × 10^−5^ M methyl jasmonate (MeJA). Relative units were normalized to the reference genes *PP2A* (for the untreated control; MS) and *UBC10*. The data represent the means ± SE (*n* = 3). Asterisks indicate significance at the * *p* < 0.05 and ** *p* < 0.01 levels. Values marked with different letters indicate significant differences between the variants based on the *t*-test (*p* < 0.05).

*GRUSP* transcripts were highly abundant in dry *Arabidopsis* seeds (ecotype Col-0) [43] compared with green siliques and 7-day-old seedlings (Figure 1A). However, within 24 h of seed imbibition, the transcript level of *GRUSP* decreased 20-fold (Figure 1B), indicating that the initial functional activity of GRUSP is associated with processes occurring in the first hours of imbibition.

In 7-day-old seedlings, where the initial mRNA level of this gene was extremely low (Figure 1A), a significant increase in *GRUSP* expression was observed only after exposure to phytohormones, such as abscisic acid (ABA; 16-fold), auxin (IAA; 5-fold), and cytokinin (tZ; 2-fold), whereas GA treatment decreased it (GA; 2-fold) (Figure 1C). The observed phytohormone-dependent expression pattern of *GRUSP* changed during plant growth. *GRUSP* was expressed at extremely low levels in rosette leaves of 3-, 5-, and 7-week-old *Arabidopsis* Col-0 plants growing in soil in the growth chamber under a 16 h light/8 h dark photoperiod at 23 °C with 100 μmol photon m^−2^ s^−1^. However, in 3-week-old plants, expression of *GRUSP* was almost completely repressed, as insensitivity to most of the phytohormones studied was observed (Figure S1). Transcription of *GRUSP* was reactivated in 5-week-old plants at a stage corresponding to the onset of first inflorescence formation, flowering, and silique formation. Then, the expression was significantly upregulated in senescence plants (7-weeks-old), whose developmental stage coincided with senescence and seed maturation (Figure S1).

### 2.2. Characterization of Grusp T-DNA Alleles

To evaluate GRUSP function, we characterized transgenic lines for the *GRUSP* gene available at the Nottingham Arabidopsis Stock Centre (NASC). According to NCBI data, the *GRUSP* gene (*At3g58450*) is 1165 bp long, consists of four exons and three introns, and encodes two mRNA variants that differ in the splicing of the first intron (Figure S2A). Genotyping the GABI-kat transgenic lines, *grusp-115*, *grusp-562*, and *grusp-756*, indicated that the T-DNA insertion for *grusp-115* is located in the 3′-untranslated (UTR) region of *GRUSP*; for the *grusp-562* transgenic line, 75 bp upstream of the start ATG codon; and for *grusp-756*, in the first intron (Figure S2A). Expression analysis of *GRUSP* mRNA level in dry seeds revealed that only the *grusp-115* mutant exhibits no *GRUSP* transcripts, while *grusp-562* and *grusp-756* are significant knockdown alleles, as reduced levels of *GRUSP* transcripts were detected in dry seeds of both transgenic lines (Figure S2C). Moreover, the presence of a T-DNA insert in the promoter region of *grusp-562* led to changes in the growth stage-dependent expression of *GRUSP* (Figure S2D). Indeed, in contrast to Col-0, a gradual accumulation of *GRUSP* transcripts was found during imbibition in *grusp-562* seeds, which persisted into the next development stages, including seedlings and young rosette leaves. Therefore, a dual effect of the T-DNA insertion in the *GRUSP* gene was observed: based on the data obtained in dry seeds, *grusp-562* might be characterized as a knock-down mutant, whereas in seedlings, it acts as an overexpression transgenic line. Besides, these results also explain germination response of *grusp-562* seeds almost similar to Col-0 but not *grusp-115* or another leaky *grusp-756* allele (Figure S3). Most likely, in dry *grusp-562* seeds the level of *GRUSP* mRNA expression is reduced, but the functional activity is not suppressed, and it accumulates at the seedling and rosette stages.

Previous data [43] showed delayed germination and ABA sensitivity in the *grusp-115* line. We analyzed the germination of *grusp* alleles on MS medium or in the presence of 1 μM ABA. We observed that *grusp-562* germinated faster than to Col-0 for the first 24 h; however, *grusp-756* showed more pronounced differences compared with Col-0 and *grusp-562* but similar to *grusp-115* (Figure S3A). Only 3% of *grusp-756* seeds had a visible root tip, compared with 43% of Col-0 seeds and 67% of *grusp-562* seeds after 24 h of germination. In the presence of 1 µM ABA, *grusp-562* seeds germinated as Col-0, but *grusp-756* and *grusp-115* seeds displayed greater sensitivity to ABA, and less than 40% of them germinated after 2 days of growth (Figure S3B).

In this study we focused on the *grusp-115* knockout line to further evaluate GRUSP function. To assess whether the after-ripening state of seeds influences the delayed germination phenotype of the *grusp-115* allele, a similar assay was conducted on seeds of the Col-0 and *grusp-115* genotypes after one month or one year of storage at 20 °C in the dark (Figure S2). After one year of storage, *grusp-115* seeds germinated to near Col-0 levels under MS conditions (70% of seeds on day 1), in contrast to seeds germinated for one month, where only 20% of *grusp-115* seeds germinated on day 1 compared with 40% of Col-0 seeds. However, the mutant line still exhibited increased sensitivity to the presence of ABA in the medium during germination, indicating that long-term storage does not eliminate the enhanced sensitivity of mutant seeds to ABA compared to Col-0 (Figure S4).

### 2.3. Germination of Grusp-115 Seeds Shows Hypersensitivity to ABA

**Figure 2:**
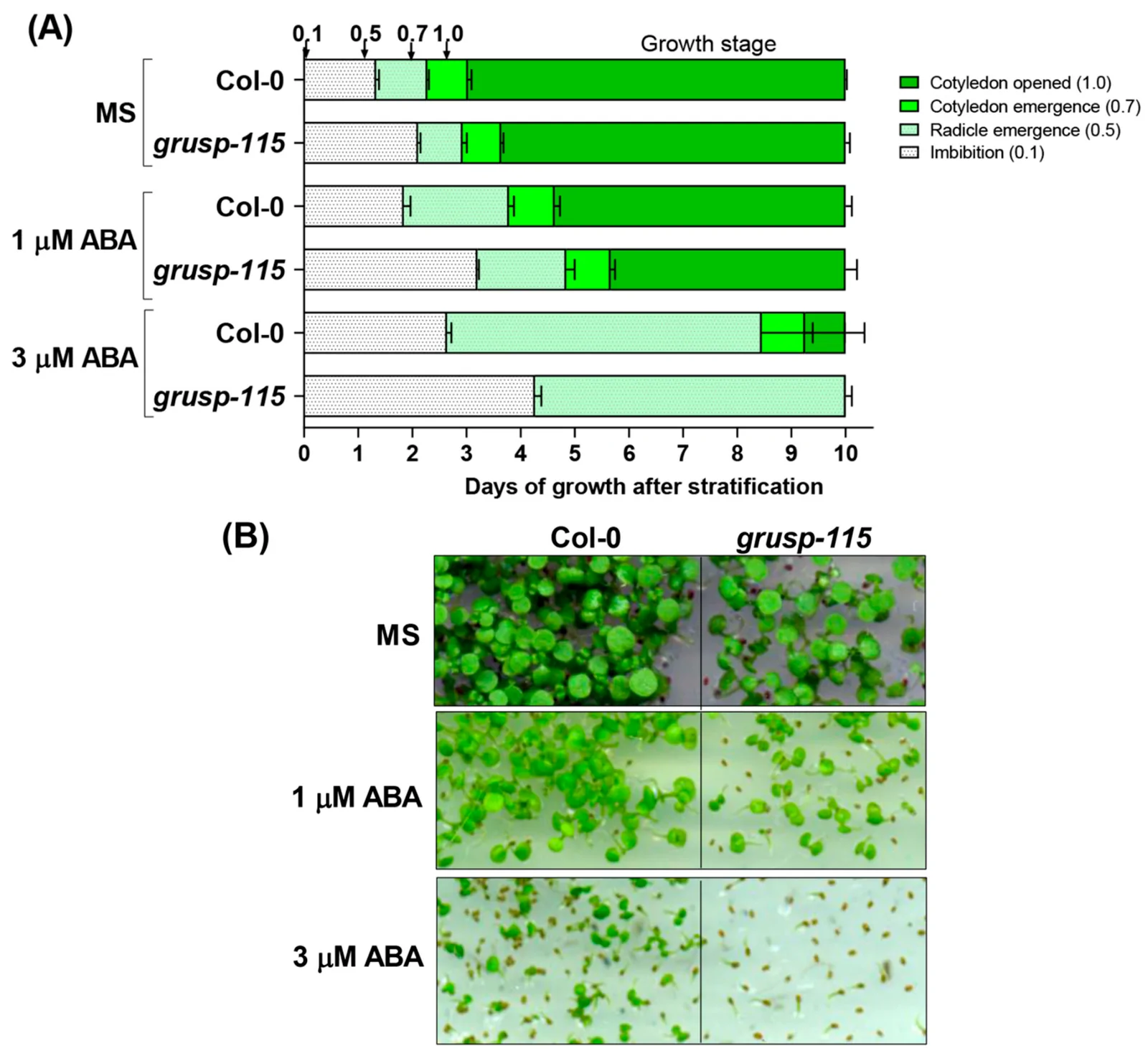
(**A**) Developmental time-course diagrams of Col-0 and *grusp-115* plants. Seeds of both lines were germinated on solid half-strength Murashige and Skoog media (MS) (control) supplemented with 1 µM or 3 µM ABA. Starting from the end of stratification and transfer of seeds to a growth chamber (day 0), seed counts were performed daily for 10 days; the indicated stages correspond to the standard classification of *Arabidopsis* developmental stages described in Boyes et al. (2001) [47]. The data represent the means ± SE. Each measurement was carried out in three biological replicates of 100 seeds per Petri dish. (**B**) Photographs of 10-day-old Col-0 and *grusp-115* seedlings grown on solid half-strength MS supplemented with or without 1 µM or 3 µM ABA; scale 5 mm.

We next tested the germination and post-germination growth of Col-0 and *grusp-115* in the presence of 1 µM and 3 µM ABA compared with 0 µM ABA (MS; control conditions) to analyze hormone sensitivity. A developmental time-course diagram for both lines over 10 days after stratification (day 0) and the transfer of seeds to the appropriate medium is shown in Figure 2. The stages correspond to the standard classification of *Arabidopsis* developmental stages described by Boyes et al. (2001) [47]. It is clearly evident that the slight delay in development of the *grusp-115* line is significantly enhanced by increasing the ABA concentration in the medium. At an ABA concentration of 3 μM, which severely affects germination, cotyledons of the *grusp-115* mutant did not appear on day 10 in this experiment, and development was arrested at the embryonic radicle stage (Boyes stage 0.5), whereas cotyledons appeared in Col-0 (Boyes stages 0.7 and 1.0) (Figure 2). As expected, *grusp-756* line showed similarity to the *grusp-115* line in developmental dynamics, while the *grusp-562* line behaved like Col-0 (Figure S5).

**Figure 3:**
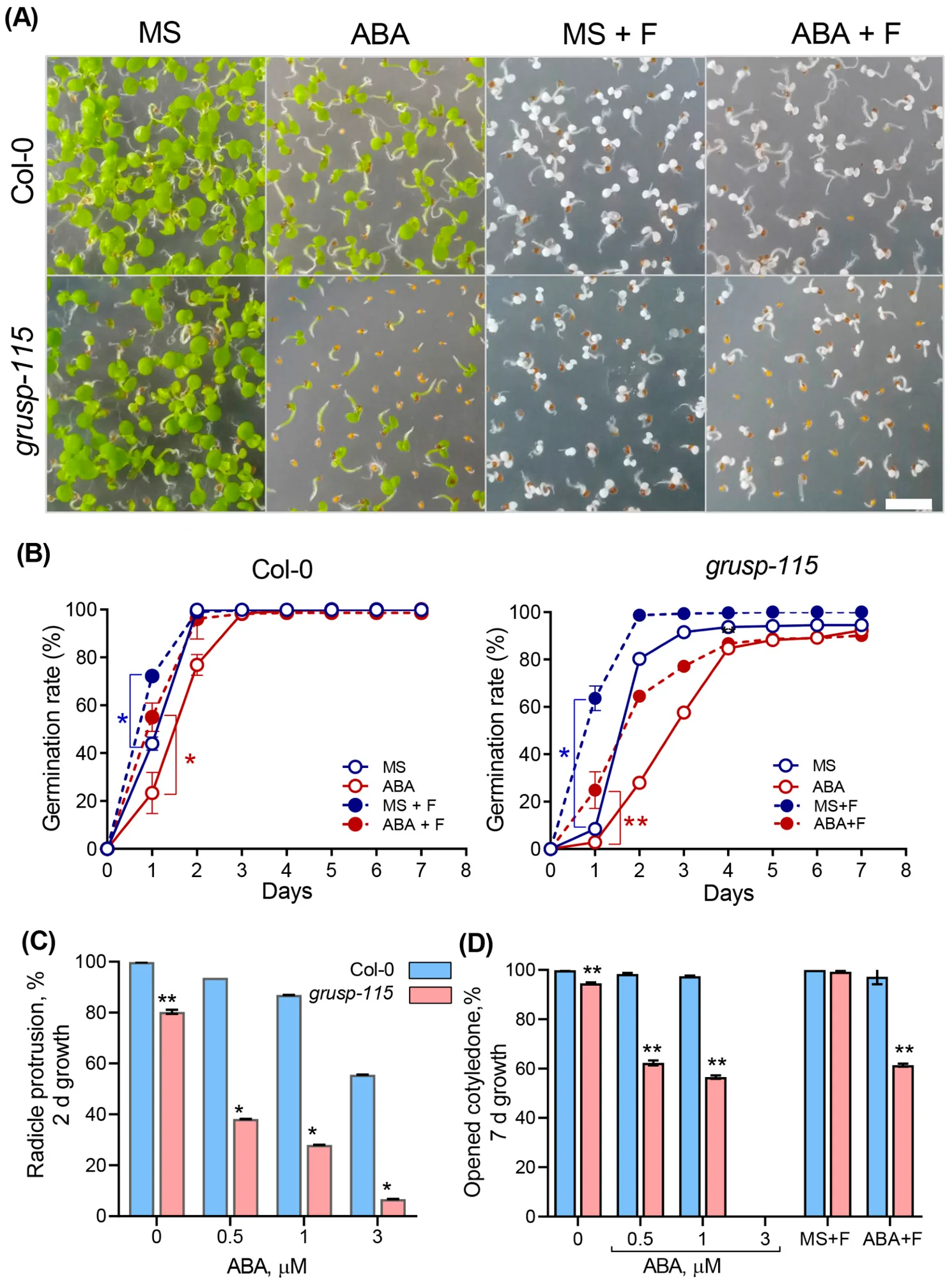
ABA germination sensitivity assay of Col-0 and *grusp-115*. (**A**) Photographs of 7-day-old Col-0 and *grusp-115* seedlings germinated on solid half-strength Murashige and Skoog media without additions (MS) or supplemented with 1 µM abscisic acid (ABA). For inhibition assays, the seeds were pretreated with 100 µM fluridone (F) for 24 h at 4 °C and then planted on corresponding plates (MS+F or ABA+F). Scale: 1 cm. (**B**) Germination rates of Col-0 and *grusp-115* seeds in the presence (ABA; red) or absence (MS; blue) of 1 µM ABA and with or without pretreatment of the seeds with 100 µM fluridone (ABA+F or MS+F, respectively). Time scale indicates the days after stratification. Radicle emergence was used as a morphological marker for germination. Each data point represents the means ± SE (*n* = 3), with 100 seeds used for each value. Asterisks indicate statistically significant differences between MS (blue) and ABA (red) fluridone-treated and untreated seeds within each genotype at the * *p* < 0.05 and ** *p* < 0.01 levels. (**C**) Diagram of radicle protrusion of 2-day-old seedlings germinated in the presence of various concentrations of ABA. (**D**) The number of expanded cotyledons in 7-day-old plants under normal conditions (0) and in the presence of ABA (0.5 µM, 1 µM, and 3 µM) or with 100 μM fluridone pretreatment without (MS+F) or in the presence of 1 µM ABA (ABA+F). Each measurement was performed in three biological replicates with 100 seeds per dish. Asterisks indicate statistically significant differences between the mutant and Col-0 plants at the * *p* < 0.05 and ** *p* < 0.01 levels.

The sensitivity to different concentrations of ABA was particularly noticeable during the first 72 h after the transfer of the seeds to light (Figure 3B and Figure S6). In the control MS plates, only 8% of the *grusp-115* seeds had a visible root tip, whereas 43% of the Col-0 plants exhibited a visible root tip 24 h after transfer to germination conditions (Figure 3B and Figure S6). This delay persisted during the subsequent 3 days of growth in the climate chamber but later disappeared. After 7 days, these differences diminished (Figure 3A,C), and the *grusp-115* mutant displayed 95% of expanded green cotyledons compared with 100% in Col-0 plants (Figure 3D and Figure S6). The presence of ABA enhanced the difference in seed germination between Col-0 and *grusp-115*. As expected, a significant decrease in germination was observed in both lines with the increase in ABA concentration (Figure 3 and Figure S6). Although nearly 80–90% of the Col-0 seeds germinated after 48 h of growth on MS medium supplemented with 0.5 μM and 1 μM ABA (Figure 3C and Figure S6), the germination percentage of the *grusp-115* seeds ranged between only 10 and 40% (Figure 3C). On the seventh day after germination, half as many expanded cotyledons were observed in the mutant line as in Col-0 under 0.5 μM or 1 μM ABA treatments (Figure 3D). Moreover, 8–9% of the *grusp-115* seeds failed to germinate during the evaluated period. In the presence of 3 µM ABA, cotyledons of germinating seeds across all the lines opened more slowly than at lower concentrations. Only a small number of Col-0 seedlings possessed fully expanded green cotyledons (1–6%), whereas the *grusp-115* mutant failed to turn green by the seventh day of growth. Ultimately, 37% of the *grusp-115* seeds failed to germinate in the presence of 3 µM ABA (Figure 3D).

**Figure 4:**
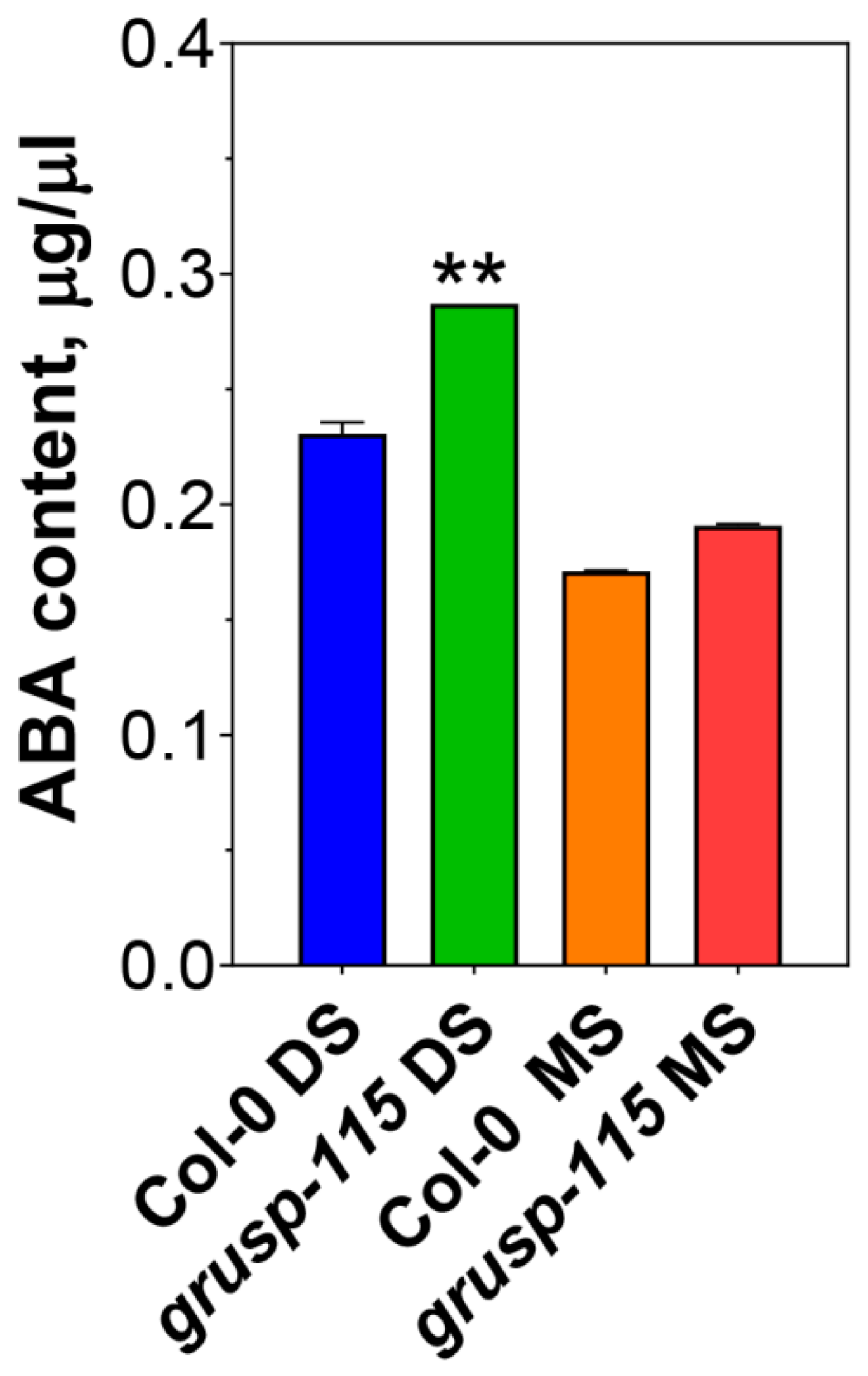
ABA quantification in Col-0 and *grusp-115* dry and 24 h MS-imbibed seeds. ABA content was determined in dry (DS) and imbibed (MS) Col-0 and *grusp-115* mutant seeds using an ELISA quantification kit (AS20 4392, Agrisera, Vännäs, Sweden). Approximately 0.5 g of freeze-dried, pulverized material obtained from dry seeds or seeds imbibed in Murashige and Skoog medium (MS) for 24 h at 23 °C was solubilized in 4.5 mL of extraction buffer. Extracts were incubated overnight in the dark at 4 °C and then centrifuged; the resulting supernatants were used for ELISA according to the manufacturer’s instruction (Agrisera). Asterisks (**) indicate significance at the *p* < 0.01 level according to the *t*-test.

The observed increase in the ABA sensitivity of *grusp-115* seeds (Figure 3C,D and Figure 4) could be attributed to alterations in tissue ABA levels. The endogenous ABA content was determined in dry and imbibed seeds using an enzyme-linked immunosorbent assay (ELISA) kit (Agrisera, Vännäs, Sweden). We found that the ABA level in the dry *grusp-115* seeds was significantly higher than in the Col-0 seeds (Figure 4). Nevertheless, after 24 h of imbibition at 23 °C, a decrease in endogenous ABA levels was observed in both Col-0 and *grusp-115* seeds, rendering their ABA contents comparable and indicating that the mutant seeds successfully catabolized their initial ABA pools (Figure 4).

To test whether the endogenous level of ABA in dry seeds causes the delayed germination phenotype of *grusp-115* seeds, the effect of fluridone (F)—an inhibitor of carotenoid biosynthesis that consequently blocks ABA biosynthesis and reduces sensitivity to ABA [48]—was also examined. In these assays, dry seeds were pretreated with 100 μM fluridone for 24 h, followed by repeated washing with distilled water. Germination was then monitored on MS medium, either with or without the addition of 1 μM ABA, to assess the germination rate relative to Col-0 as described by Chiriotto et al. (2023) [49].

As expected, the seedlings of both genotypes obtained from fluridone-treated seeds were white [48] (Figure 3A; MS+F and ABA+F images). Pretreatment with fluridone significantly improved seed germination in both genotypes on control MS or ABA-supplemented media (Figure 3B,D). After 24 h of germination, the percentage of germinated Col-0 seeds pretreated with fluridone was nearly 30% greater than that of untreated seeds (Figure 3B; Col-0). A similar but more pronounced effect was observed in *grusp-115*: fluridone pretreatment increased the percentage of germinated seeds by almost 60% (Figure 3B; *grusp-115*), and the number of expanded cotyledons in the pretreated (MS+F) seeds became similar to that of Col-0 by day 7 of growth (Figure 3D).

Furthermore, fluridone also reduced the pronounced inhibition of Col-0 and *grusp-115* seed germination induced by exogenous ABA, although inhibition remained significantly greater in the mutant, which did not achieve Col-0 germination rates (Figure 3B). After 24 h of germination, nearly 55% of pretreated Col-0 seeds exhibited radical emergence on medium supplemented with 1 μM ABA, compared with approximately 23% of the untreated seeds; after two days, nearly 100% of the pretreated seeds had germinated (Figure 3B).

Fluridone improved the germination of *grusp-115* seeds on ABA-supplemented medium (ABA+F) by nearly 20% compared with untreated seeds; however the mutant still retained a predominance of slow-germinating or arrested seeds (Figure 3B). This trend was particularly noticeable during the cotyledon emergence stage by the seventh day of germination (Figure 3D). Col-0 seedlings developed fully expanded cotyledons on media both with and without ABA. In contrast, in the germinating *grusp-115* line, cotyledons emerged in only 60–65% of the seedlings, and pretreatment with fluridone only marginally increased this percentage. Thus, even in the presence of an ABA biosynthesis inhibitor, the *grusp-115* mutant still exhibited a more pronounced response to exogenous ABA compared with Col-0.

We also evaluated effect of ABA and fluridon on lines with knockdown alleles: the *grusp-756* line showed the appearance of opened cotyledons at a similar rate to *grusp-115*, demonstrating ABA-hypersensitivity that was only partially rescued by fluridone, whereas *grusp-562* behaved similarly to Col-0 (Figure S7).

### 2.4. Changes in the Expression of Genes Related to ABA Metabolism in the Grusp-115 Mutant

**Figure 5:**
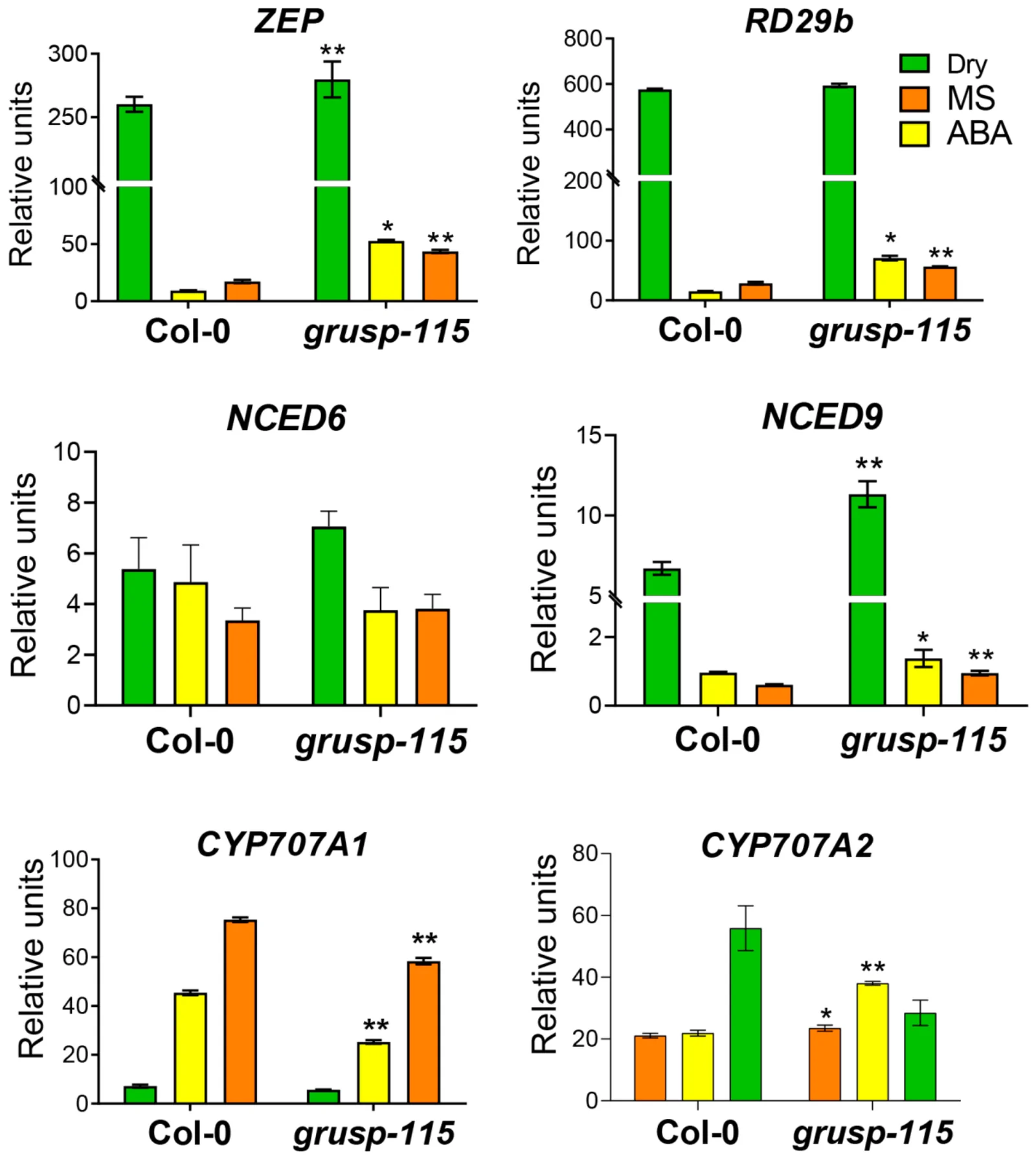
Expression patterns of genes related to ABA metabolism in Col-0 and *grusp-115* seeds. Relative expression levels of ABA biosynthetic (*ZEP*, *NCED6*, and *NCED9*), catabolic (*CYP707A1* and *CYP707A2*), and ABA-responsive (*RD29b*) genes were analyzed by qRT-PCR in dry seeds (DS) and seeds incubated for 24 h on Murashige and Skoog (MS) medium or with 50 μM ABA under a 16 h light/8 h dark photoperiod at 23 °C with 100 μmol photons m^−2^ s^−1^. *UBC10* was used as the internal reference gene for qRT-PCR. Data represent the means ± SE (*n* = 3). Asterisks indicate statistically significant differences between Col-0 and the *grusp-115* mutant at the * *p* < 0.05 and ** *p* < 0.01 levels based on Student’s *t*-test.

To understand the molecular basis of the altered seed response to ABA, we next assessed the expression levels of key genes involved in ABA metabolism. We next assessed the expression levels of key genes involved in ABA metabolism (Figure 5) using qRT-PCR analysis. Transcript levels were determined in 3-month-old dry seeds of the *grusp-115* mutant compared with Col-0, alongside changes in mRNA abundance in seeds imbibed for 24 h in either MS medium or a 50 µM ABA-supplemented solution (Figure 5).

The regulation of ABA levels in seeds is carried out through three main synthesis and metabolic steps controlled by zeaxanthin epoxidase (ZEP), two isoforms of 9-cis-epoxycarotenoid dioxygenase NCED (NCED6 and NCED9) and an isoform of cytochrome P450-dependent ABA 8′-hydroxylase (CYP707A1-3) [7]. As shown in Figure 5, the *ZEP* mRNA level was reliably greater in the dry *grusp-115* seeds than in the Col-0 seeds. The increase in *NCED9* expression was similar, but the difference in *NCED6* expression was not significant between the *grusp-115* and Col-0 groups (Figure 5).

**Figure 6:**
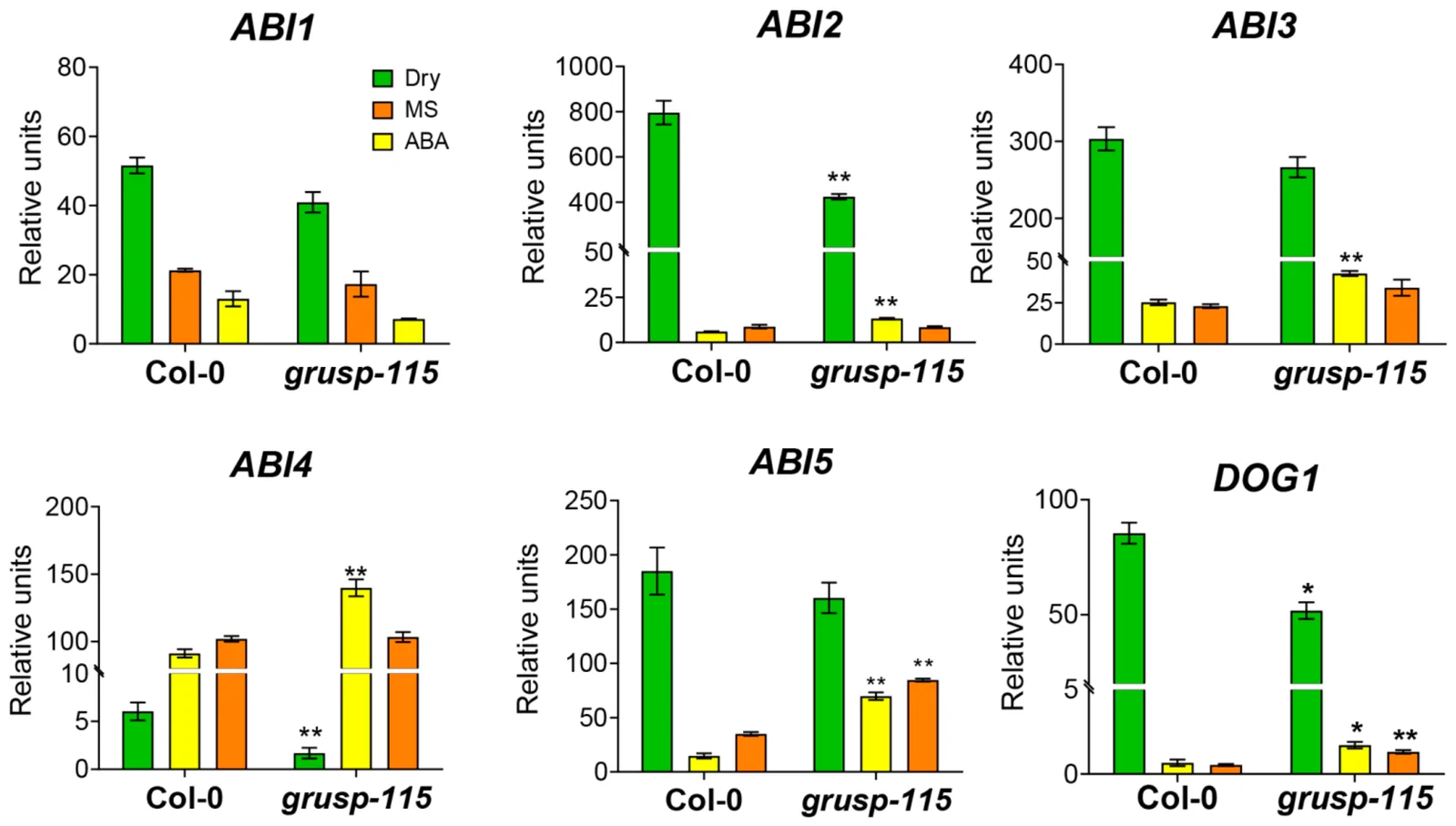
Expression patterns of genes related to ABA signaling in Col-0 and *grusp-115* seeds. The relative expression levels of genes were analyzed using qRT-PCR in dry seeds (DS) and seeds incubated for 24 h on Murashige and Skoog (MS) medium or with a 50 μM ABA solution under a 16 h light/8 h dark photoperiod at 23 °C with 100 μmol photon m^−2^ s^−1^. Relative units were normalized to the reference gene *UBC10*. Data represent the means ± SE (*n* = 3). Asterisks indicate statistically significant differences between Col-0 and the *grusp-115* mutant at the * *p* < 0.05 and ** *p* < 0.01 levels based on Student’s *t*-test.

The decrease in the level of ABA in germinating seeds and young seedlings is caused by the high activity of *CYP707A2*, the main enzyme involved in ABA catabolism [9]. Compared with that in Col-0, the expression of *CYP707A2* in dry *grusp-115* seeds was reduced (Figure 5), which is consistent with a higher ABA level in dry mutant seeds than in Col-0 seeds (Figure 4). This gene is characterized by an intricate expression profile during 24 h of imbibition and depends on the ABA level [9]. As shown in Figure 6, the *CYP707A2* mRNA level was reduced twofold in Col-0 seeds imbibed for 24 h compared with dry seeds. In contrast, compared with that in dry seeds, *CYP707A2* expression in imbibed *grusp-115* seeds was even greater. Like the expression of *CYP707A2*, the expression of *RD29b*, an ABA marker, sharply decreased in the seeds of both genotypes after 24 h of imbibition; however, the expression of *RD29b* was fourfold greater in imbibed *grusp-115* seeds. Meanwhile, the *RD29b* gene was not induced in ABA-treated seeds of the *grusp-115* mutant (Figure 5) compared with MS-imbibed seeds, indicating that the response of this ABA marker gene is close to saturation as a result of the increased endogenous ABA level (Figure 4) in dry mutant seeds compared with that in Col-0 seeds.

Similarly, transcripts of the ABA biosynthetic enzyme *ZEP* were strongly downregulated within 24 h of imbibition in both genotypes but were still more highly expressed in the imbibed *grusp-115* seeds than in Col-0 seeds (Figure 5). In addition, the expression of *CYP707A1* was twofold lower in the imbibed mutant seeds than in the Col-0 seeds (Figure 5).

### 2.5. Altered ABA Signaling in the Grusp-115 Mutant

Germination induction not only depends on the internal ABA level but is also regulated by the sensitivity of seed tissues to ABA. Therefore, we examined the expression levels of genes associated with ABA signaling and involved in the ABA-dependent regulation of seed dormancy and germination (Figure 6 and Figure S8). In dry seeds, no significant differences in the expression of the *ABI1*, *ABI3*, and *ABI5* genes were observed between Col-0 and *grusp-115* (Figure 6). However, the transcript level of the *ABI2* gene, encoding a signaling phosphatase from the *PP2C* group, was reduced in *grusp-115* dry seeds compared with that in Col-0 seeds (Figure 6). Expectedly, the *ABI4* transcript level was lower in dry seeds compared with the other studied genes; however, in the *grusp-115* mutant, its level was fourfold lower than in Col-0. The transcript level of the *DELAY OF GERMINATION 1* (*DOG1*) gene, which is not involved in the ABA signaling pathway but plays an important role in the maintenance of ABA-dependent seed dormancy [50], was reduced in dry *grusp-115* seeds (Figure 6).

After 24 h of seed imbibition, all the genes involved in ABA signaling, with the exception of *ABI4*, were downregulated, which is necessary for the inactivation of ABA-dependent responses and the initiation of germination (Figure 6). However, this downregulation occurred to a lesser extent in imbibed mutant seeds than in Col-0, resulting in significantly higher transcript levels after 24 h. Such a pattern of expression was observed for *ABI2*, *ABI3*, *ABI5*, and *DOG1*, which is consistent with the positive regulation of these genes by endogenous ABA at the germination stage. *ABI1* gene expression was indistinguishable between *grusp-115* and Col-0 (Figure 6), indicating that GRUSP does not act via *ABI1*.

In contrast, *ABI4* expression was upregulated after 24 h of imbibition in both lines, despite a decrease in ABA levels. In *grusp-115*, this activation was even greater, resulting in a higher transcript level after 24 h of imbibition, although *ABI4* expression was fourfold lower in dry mutant seeds than in Col-0 (Figure 6).

### 2.6. ABA Hypersensitivity in Grusp-115 Is Associated with GA Metabolism

**Figure 7:**
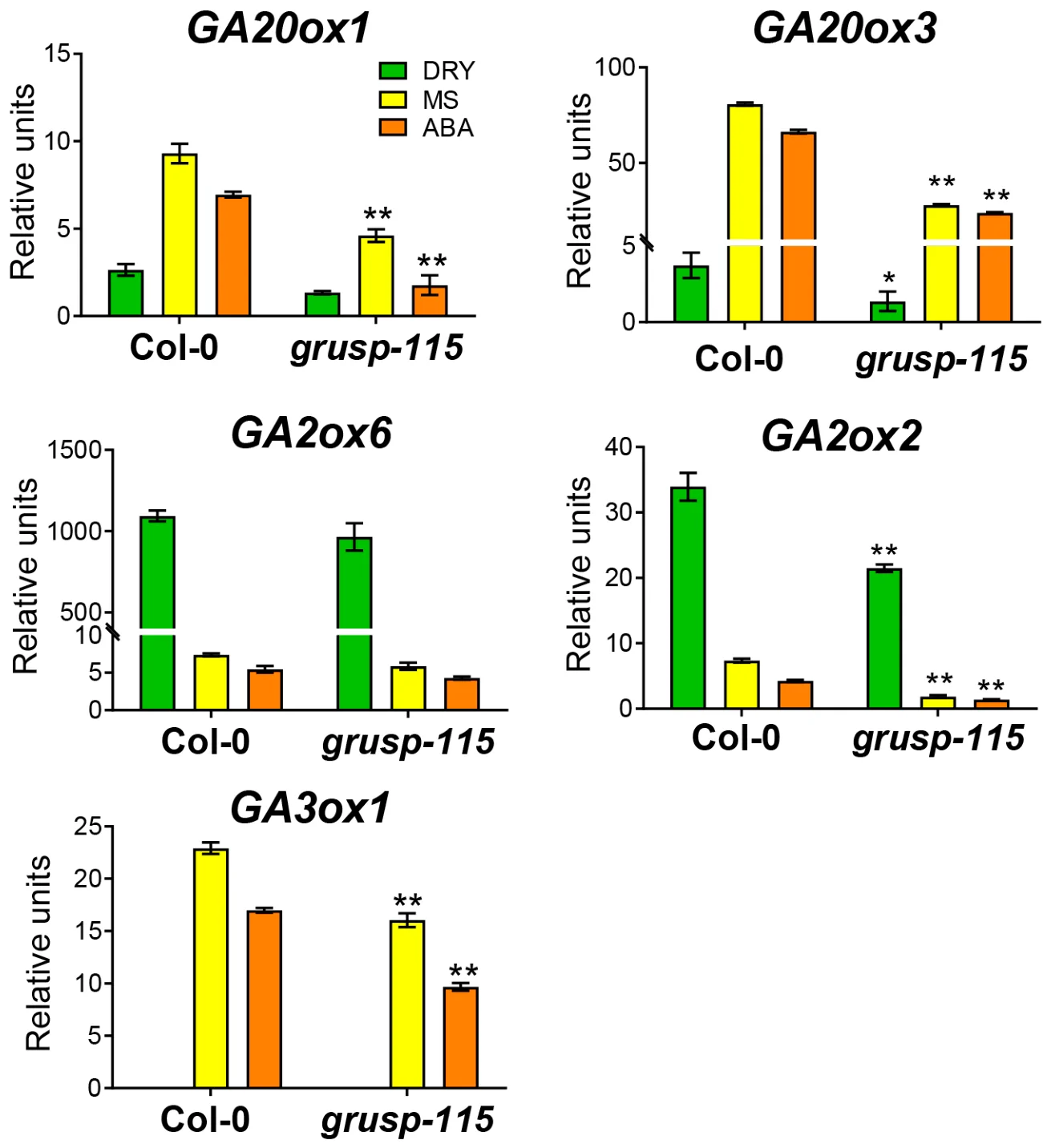
Expression patterns of genes associated with GA metabolism in Col-0 and *grusp-115* seeds. The relative expression levels of genes were analyzed by qRT-PCR in dry seeds (DS) and seeds incubated for 24 h on Murashige and Skoog (MS) medium or a 50 μM under a 16 h light/8 h dark photoperiod at 23 °C with 100 μmol photon m^−2^ s^−1^. Relative units were normalized to the reference gene *UBC10*. Data represent the means ± SD (*n* = 3). Asterisks indicate statistically significant differences at the * *p* < 0.05 and ** *p* < 0.01 levels between Col-0 and the *grusp-115* mutant (*t*-test).

The *grusp-115* mutant demonstrated an altered content of bioactive GAs in rosette leaves [45] (Table S2) and downregulation of the basal mRNA levels of *Ga20ox* biosynthetic genes [44]. Since gibberellins act as antagonists of ABA during germination, the ABA hypersensitivity phenotype of *grusp-115* may also be associated with disturbances in either the metabolism or signaling pathway of GAs that extend to vegetative stage. In this regard, the mRNA levels of genes associated with GA metabolism in seeds were analyzed. The transcript levels of the *GA20ox1*, *GA20ox3*, and *GA3ox1* genes, which are responsible for the final stages of gibberellin biosynthesis, were upregulated upon imbibition (Figure 7). However, their expression levels were reduced in the imbibed seeds of the *grusp-115* mutant compared with those of Col-0 (Figure 7). Moreover, the expression of not only the genes encoding GA biosynthetic enzymes but also the *GA2ox2* gene encoding a catabolic enzyme is reduced in both dry and imbibed mutant seeds. For other genes encoding GA catabolic enzymes, such as *GA2ox6*, no significant differences were detected between Col-0 and the *grusp-115* mutant line (Figure 7). These results indicate that loss of GRUSP function results in reduced expression of genes related to gibberellin metabolism, which contributes to delayed germination.

### 2.7. Loss of GRUSP Influence on Seedling Growth

#### 2.7.1. The Response of the *Grusp-115* Mutant to ABA Application During Primary Root Growth

Exogenous ABA strongly inhibited the germination of *grusp-115* seeds (Figure 2 and Figure 3). Furthermore, we previously demonstrated that the *grusp-115* exhibits developmental delays, resulting in postponed flowering [45].

Given that ABA is a key regulator of stress responses and growth retardation, we next analyzed whether this delay in vegetative development is associated with impaired hormonal signaling. To evaluate post-germination growth we analyzed the effect of ABA on primary root elongation in *grusp-115* mutant compared to Col-0. The dose-dependent effect of ABA on primary root elongation was assessed either in 10-day-old seedlings grown directly on vertical agar plates without (control) or with ABA, or in 10-day-old seedlings after their transfer at day 4 of growth to half-strength MS media with increasing hormone concentrations (0.1 μM; 0.25 μM; 0.5 μM; 0.75 μM, and 3 μM).

**Figure 8:**
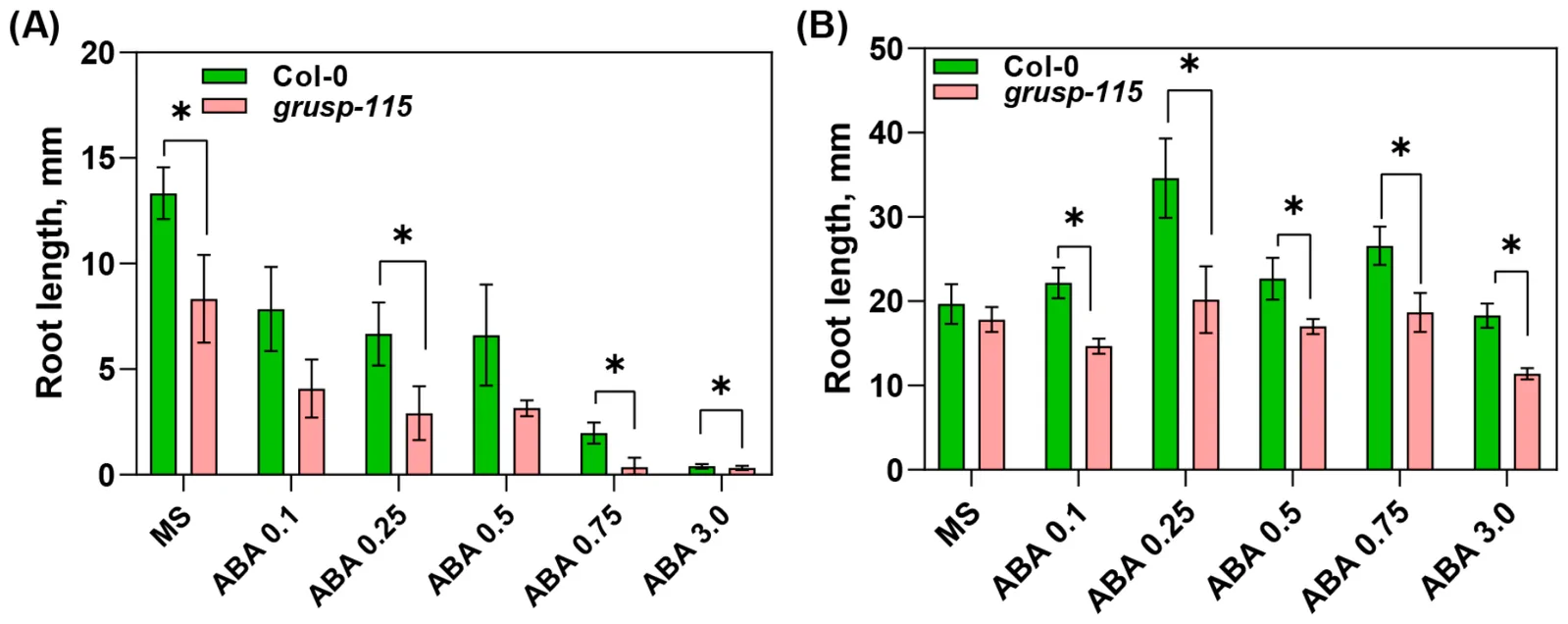
Primary root lengths of Col-0 and *grusp-115* seedlings in the presence of abscisic acid (ABA). (**A**) Root lengths of 10-day-old seedlings grown directly on vertical agar plates with half-strength Murashige and Skoog medium supplemented with a range of ABA concentrations (0.1 µM–3 µM) or without the hormone (MS). (**B**) Four-day-old seedlings with uniform root lengths were transferred to vertical MS agar plates with or without 0.1 µM–3 µM ABA, and the primary root length was measured 6 days after transfer. Data represent the means ± SE. Each measurement was carried out in two biological replicates with 25 seedlings per plate for each line. Asterisks indicate statistically significant differences based on one-way ANOVA followed by Student’s *t* test at the level of *p* < 0.05.

Direct germination under control conditions (no hormone addition) and at the ABA concentration (0.1 μM) showed, respectively, a slightly shorter or similar primary root length in the *grusp-115* mutant compared to Col-0 on the tenth day of germination (Figure 8A). In the presence of increased hormone concentrations (0. 25 µM to 3 µM ABA), retarded primary root development was more pronounced in the *grusp-115* line (Figure 8A), due to the delayed germination of mutant seeds.

Following the transfer of 4-day-old Col-0 seedlings to ABA-supplemented media, a low concentration of ABA (0.25 μM) exerted a highest stimulating effect on primary root elongation after 6 days of growth in our experimental conditions. In contrast, for *grusp-115*, showed a rather neutral or suppressed response to ABA even after transfer to low dose of ABA (0.1 μM and 0.25 μM), and the root length was significantly shorter than in Col-0, indicating that *grusp-115* exhibits an altered growth response compared to Col-0 under the selected ABA treatment conditions. With a further increase in the ABA concentration, stimulation of primary root growth at 0.5 and 0.75 μM was not observed in mutant seedlings. At the inhibitory ABA concentration (3 μM), the suppression of primary root elongation was clearly visible in both genotypes but was again more pronounced in the *grusp-115* mutant (Figure 8B), indicating a dose-dependent response and a phenotype of hypersensitivity to ABA at the stage of seedling development.

#### 2.7.2. The *Grusp-115* Seedlings Demonstrates Enhanced Expression of ABA Signaling Genes

**Figure 9:**
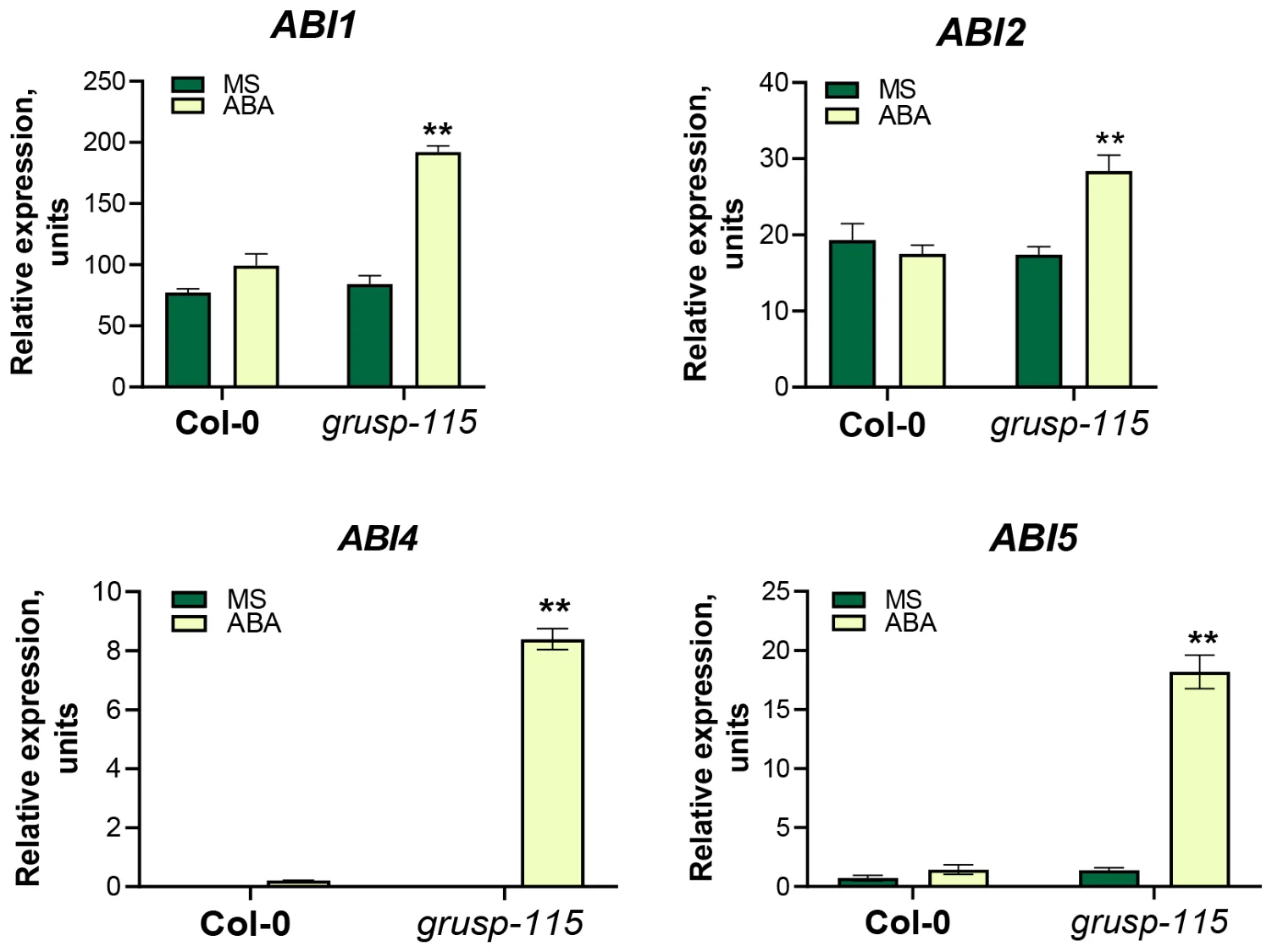
Expression analysis of genes associated with ABA signaling in 7-day-old seedlings of *grusp-115* and compared to Col-0. Seedlings were grown on Murashige and Skoog (MS) medium or the presence of 1 μM ABA under a 16-h light/8-h dark photoperiod at 23 °C with 100 μmol photon m^−2^ s^−1^. Relative units were normalized to the reference gene *UBC10*. Data represent the means ± SEs (*n* = 3). Asterisks indicate statistically significant differences at ** *p* < 0.01 between the Col-0 and the *grusp-115* mutant (*t* test).

Because *grusp-115* mutant seedlings exhibited hypersensitivity to low doses of ABA during root growth (Figure 8B) in addition to altered germination (Figure 3), we analyzed the expression of key ABA-signaling genes, *AB1/2/4/5* in 7-day-old seedlings grown under standard conditions (MS) or supplemented with ABA (1 μM). As shown in Figure 9, on MS medium, expression levels in mutant seedlings were almost similar to Col-0 that differs from that expression in MS-imbibed seeds, indicating that endogenous ABA level is comparable in seedlings of both lines. However, in the presence of ABA, a sharp upregulation expression of *ABI1*, *ABI2*, and especially *ABI4* and *ABI5* was observed in *grusp-115* line indicating that the ABA signal perception and transduction system in the mutant is in a “hyperactive” state.

#### 2.7.3. *Grusp-115* Seedlings Exhibit Excessive Accumulation of ABI5 Protein upon GA

The transcription factor ABI5 protein (ABSCISIC ACID INSENSITIVE5 (ABI5)) is one of the major regulatory proteins involved in seed germination [51] and post-germination growth, including flowering [52]. Given the reduced accumulation of *GRUSP* mRNA in *abi5* mutant seeds [26], as well as upregulation of *ABI5* transcript in *grusp-115* seedlings, we hypothesized a functional relationship between these proteins.

**Figure 10:**
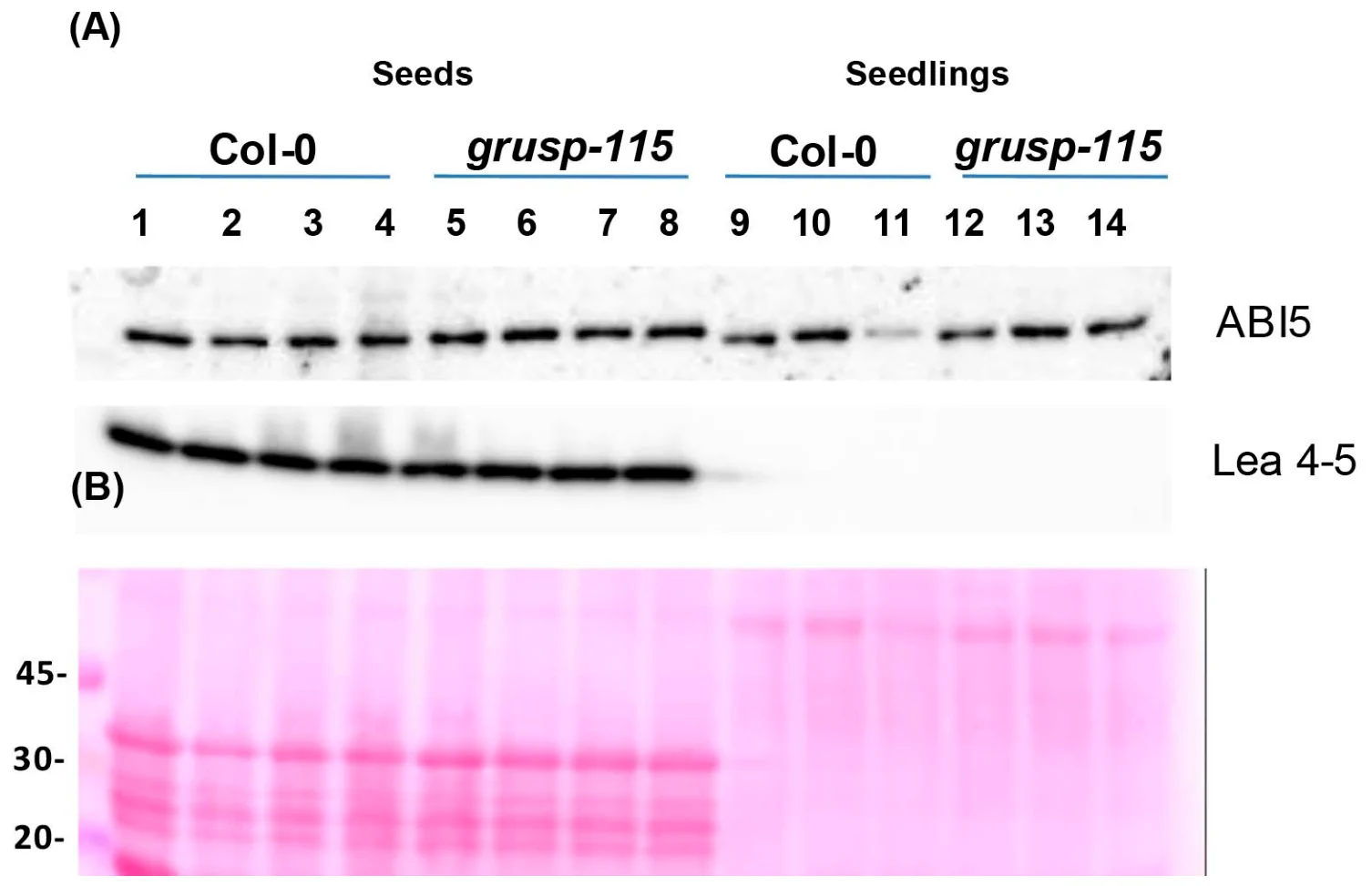
ABI5 protein levels in response to exogenous applied gibberellins (GAs) and paclobutrazol (PAC) in Col-0 and *grusp-115* seedlings. Total protein extracts were prepared from seeds and 10-day-old seedlings soaked in the presence of 10^−5^ M GA and 5 × 10^−6^ M PAC or without additions (MS). (**A**) Samples with equal amounts (25 µg of total protein) were analyzed by western blotting using antibodies specific for ABI5 (Agrisera). Lea 4-5 (Agrisera) protein was used as loading control for seed total protein extracts. (**B**) Equal protein loading was checked by staining of blots with Ponceua S. Numbers at the left of blot indicate the molecular weights of standard proteins in kDa. Designations: 1, 5—dry seeds; 2, 6—3 h MS-imbibed seeds; 3, 7—3 h PAC-imbibed seeds; 4, 8—3 h GA-imbibed seeds; 9, 12—3 h MS-treated seedlings; 10, 13—3 h PAC-treated seedlings; 11, 14—3 h GA-treated seedlings.

Excess GA reduces ABI5 levels, while gibberellin limitation maintains high ABI5 levels. We analyzed the ABI5 protein levels in response to exogenously applied GA_s_ and paclobutrazol (PAC), an inhibitor of GA synthesis, in dry seeds, as well as in seeds and 10-day-old seedlings soaked for 3 h in liquid MS or MS supplemented with GA_4+7_ or PAC. The analysis showed that in dry seeds (Figure 10) the ABI5 level was comparable in both genotypes. In our experimental conditions, during 3 h of imbibition on MS medium, a slight decrease in the ABI5 level was observed in the Col-0 seeds, while it remained higher in the *grusp-115* mutant seeds. In contrast to the mRNA levels of *ABI5* (Figure 6), the three-hour treatment of seeds with GAs and PAC did not influence ABI5 protein level in both genotypes. However, in the 10-day-old seedlings, excess GAs sharply dropped the ABI5 protein level in Col-0 but stayed constant in the mutant. Under GA-deficient conditions (PAC), its accumulation was similar to the Col-0. These data demonstrate that *GRUSP* deficiency results in the stabilization of ABI5 protein level under GAs-enhanced conditions and renders it resistant to the repressive action of GA, starting from seed germination and continuing during seedling growth.

## 3. Discussion

The functions of USPs are primarily associated with the development of plant resistance to different stress conditions [33,34,35,36,37]; however, the properties of the USP domain are typical for many regulatory proteins [29]. This fact allows us to consider the participation of proteins with a USP domain in a wide variety of different signaling pathways that are not necessarily related to stress.

Indeed, our results for the *At3g58450* gene, encoding a putative USP-like protein, revealed a functional link between germination-related universal stress protein (GRUSP) and mechanisms that may control seed germination and post-germination growth. Expression analysis demonstrated the greatest accumulation of *At3g58450* mRNA in dry seeds [43], which rapidly decreased within 24 h upon imbibition (Figure 1A,B). *GRUSP* expression is induced by ABA and auxin (AUX) while repressed by GAs (Figure 1C). Similar expression patterns were shown for several transcription factors, FUSCA3 (FUS3), ABI3, LEC1, and LEC2, whose mRNA levels were abundant during embryogenesis and decreased upon imbibition (summarized in Chiu et al. 2012 [53]). The function of these transcription factors is associated with ABA, GA, or AUX biosynthesis and signalling pathways, and their repression/activation during seed imbibition is required to initiate vegetative growth [3,46].

Loss of *GRUSP* function led to a deficiency of bioactive gibberellins [45] (Table S2). This phenotype is manifested by a decrease in the basal mRNA level of *Ga20ox* biosynthetic genes, a decrease in sensitivity to exogenous GAs, and the accumulation of the DELLA family GAI (Gibberellic Acid Insensitive) protein [44]. In addition, the mutant exhibited a delay in flowering under long-day conditions, a reduced pool of endogenous bioactive GA1 and GA3 (Table S2), and an increased expression level of the flowering repressor FLC (Flowering Locus C) [45]. Here we showed that dry *grusp-115* mutant seeds were characterized by an increased ABA level (Figure 4) and more susceptible to ABA at germination and post-germination growth (Figure 2, Figure 3 and Figure S5), indicating the involvement of GRUSP in processes co-regulated by both GAs and ABA. Thus, the mutation caused a pleiotropic effect, demonstrating that the mutant’s hormonal balance is shifted toward repression of GAs responses or, conversely, higher activation of ABA responses.

An increased endogenous ABA content in the dry mutant seeds was accompanied by the upregulation of ABA biosynthetic genes and the downregulation of ABA catabolism genes, indicating that the mutation in the *GRUSP* gene influences the metabolism of ABA. Consistent with this, *grusp-115* dry seeds exhibited increased accumulation of transcripts for the ABA biosynthesis genes *ZEP*, *NCED9*, alongside reduced mRNA levels of the catabolic gene *CYP707A2* (Figure 6). Conversely, expression of *NCED6*, another key gene of ABA biosynthesis, remained unchanged in seeds; whereas the catabolic gene *CYP707A1* showed a minor upregulation in *grusp-115* compared Col-0 seeds. Given that *CYP707A2* is important during early germination and promotes degradation of ABA and dormancy release [9], the reduction in its mRNA levels in the mutant line could potentially contribute to the observed germination delay.

Next, mutant seeds showed hypersensitivity to exogenously apply ABA, and their pretreatment with fluridone (an inhibitor of ABA synthesis) did not reduce ABA sensitivity (Figure 3). The observed hypersensitivity to ABA was not limited to germination but persisted throughout of seedling development: transplanted seedlings exhibited delayed root growth already (Figure 8). When seedlings have already formed, the stimulating effect of ABA on root growth is weak. This is indicated by the fact that 0.25 μM ABA suppresses root growth. Root elongation by exogenous ABA in *Arabidopsis* is biphasic and occurs through various pathways involving other phytohormones [54]. The stimulatory effect of low ABA concentrations (up to 1 μM ABA) on root growth occurs through an ethylene-independent pathway and requires auxin signalling and auxin transport [54,55]. The absence of a stimulatory effect of ABA relative to Col-0 at concentration 0.1 μM or 0.25 μM ABA indicates the hypersensitivity of the mutant or the involvement of GRUSP in different pathways of ABA acting through other phytohormones [55,56,57,58]. Our study indicates that defects in *GRUSP* expression may affect other hormonal pathways. The statistically significant increase in the level of the *GRUSP* transcript in response to a set of exogenous hormones suggests that, in addition to ABA and GA [44], this protein is likely involved in the auxin, cytokinin, brassinosteroids, ethylene, and salicylic acid pathways (Figure 1C and Figure S1). Therefore, the dormancy-to-germination transition of mutant seeds must be considered in the context of the mutual influence of other hormonal networks.

A delay in germination may also be associated with deep dormancy, when the seeds initially emit an intense ABA signal that is more difficult to inactivate. This phenomenon is observed, in particular, in the *Arabidopsis* Cvi ecotype [59]. The deep dormancy state is characterized, among other things, by increased expression of the *DOG1* gene, which regulates the physiological dormancy of seeds [60], and the ABA-responsive genes *ABI3*, *ABI4*, and *ABI5* [7]. In our study, compared with Col-0, the *DOG1* transcript level in dry mutant seeds was significantly reduced (Figure 6). On the other hand, during imbibition, transcript levels of *DOG1* and all genes involved in ABA-dependent signaling decreased more slowly than in Col-0. In the absence of GRUSP, the initial transcript levels of ABA-dependent signaling genes in dry seeds of the *grusp-115* mutant were similar (*ABI1*, *ABI3*, and *ABI5*) or even 2-fold lower (*ABI2* and *ABI4*) than those in Col-0 (Figure 6). Also, *grusp-115* seeds 12 months after ripening germinated on MS medium comparably to Col-0 seeds (Figure S4). Therefore, the phenotype of *grusp-115* seeds is not associated with a deep dormancy state.

Therefore, although the basal ABA sensitivity in dry mutant seeds does not exceed that of Col-0, the observed ABA sensitivity in mutant seeds during imbibition, despite the suppression of endogenous ABA synthesis, indicates impairments primarily in the ABA signaling stages in the mutant, rather than directly in ABA biosynthesis, and that the ABA signaling and transduction system in the mutant is in a “hyperactive” state. This is supported by either the absence or slight induction in ABA-treated seeds or sharply upregulation in ABA-treated seedlings of ABA-signaling genes, as well as higher levels of ABI5 protein in mutant seeds soaked in MS or even after GA treatment.

The *grusp-115* phenotype closely resembles that of lines directly [16,20] or indirectly upregulating *ABI4* or *ABI5* [61,62]. Under control conditions, these overexpressing lines typically display either a subtle germination delay (like the ABI4OX line) or remain virtually indistinguishable from Col-0 (ABI5OX line), yet both exhibit profound hypersensitivity to exogenous ABA [16,20]. A parallel pattern is observed upon the loss of *MOTHER OF FT AND TFL1* (*MFT*); the *mft* knockout mutant germinates normally on MS medium but experiences a severe developmental lag in the presence of ABA, a response coupled with the hyper-induction of *ABI5*. Moreover, much like *GRUSP*, *MFT* transcription is stimulated by ABA, is repressed by GAs, and functions to suppress *ABI5* expression [61]. Furthermore, mirroring AGL21-overexpressing plants [62], *grusp-115* displays a slight germination delay, ABA hypersensitivity, and retarded cotyledon greening. This phenotypic overlap extends to the expression profiles; during imbibition, both genotypes exhibit a characteristic increase in *ABI4* and *ABI5* transcripts relative to Col-0, while *ABI1* expression remains unaltered.

High-throughput cistromic analyses, including ChIP-seq and DAP-seq, confirmed that *At3g58450* (*GRUSP*) is a target of the ABI5 transcription factor [63]. This link is highly consistent with earlier transcriptome maps by Nakabayashi et al. (2005) [24] and Reeves et al. (2011) [26], which showed that *GRUSP* expression depends on core ABA signaling regulators, including ABI5. This is consistent with the data presented in the PlantPAN5.0 database (https://plantpan.itps.ncku.edu.tw/plantpan5/, accessed 14 July 2026) on the presence of *cis*-regulatory elements for ABI4 (5′-gaCCGACcat-3′) and ABI5 (5′-tgcgtCGTCAcaatc-3′) in the promoter region of the *GRUSP* gene (Figure S9). Our data revealed that *ABI5* transcript levels were significantly elevated in *grusp-115* seeds during both imbibition and exogenous ABA treatment compared with Col-0 (Figure 6). Moreover, we also found that ABI5 protein level remains stable under GAs excess conditions in *grusp* seedlings (Figure 10), that could indirectly affect its further development.

It is known that AB5 protein turnover is regulated in a complex manner, which limits excessive accumulation of ABI5 [64]. Based on primary amino acid sequence and according to the N-end rule [65] GRUSP is predicted to be a short-lived protein (with a half-life of approximately 1 h) due to the presence of a glutamate residue (Glu; E) in penultimate position from initiating methionine (Figure S10). Domain organization analysis assigns GRUSP to the adenine nucleotide alpha hydrolases-like superfamily, suggesting its capacity to bind and/or hydrolyze adenine nucleotides [44]. Furthermore, as other USP-proteins [35] GRUSP may undergo autophosphorylation or participate in the phosphorylation of downstream target proteins. Therefore, GRUSP could functionally influence at the protein level rather than at transcription and could be involved in regulation of ABI5.

Indeed, the stability of the ABI5 protein during late development may explain the phenotype observed in *grusp-115*. It has a reduced content of bioactive gibberellins and reduced expression of the *GA20ox* genes, which correlates with an increased basal level of the GAI repressor protein. At the same time, the mutant has increased expression of the *FLC* gene [44,45]. This fact is fully consistent with the mechanisms, according to which, under conditions of GA deficiency, the GAI protein accumulates, which indirectly leads to repression of *GA20ox* [66] and activation of the flowering repressor FLC through its direct interaction with ABI5 [67], which activates *FLC* transcription in an ABA-dependent manner [52]. Thus, the ABI5 protein is one of the possible reasons linking disturbances in seeds with subsequent delays in the development and flowering of an adult plant of *grusp-115* mutant.

**Figure 11:**
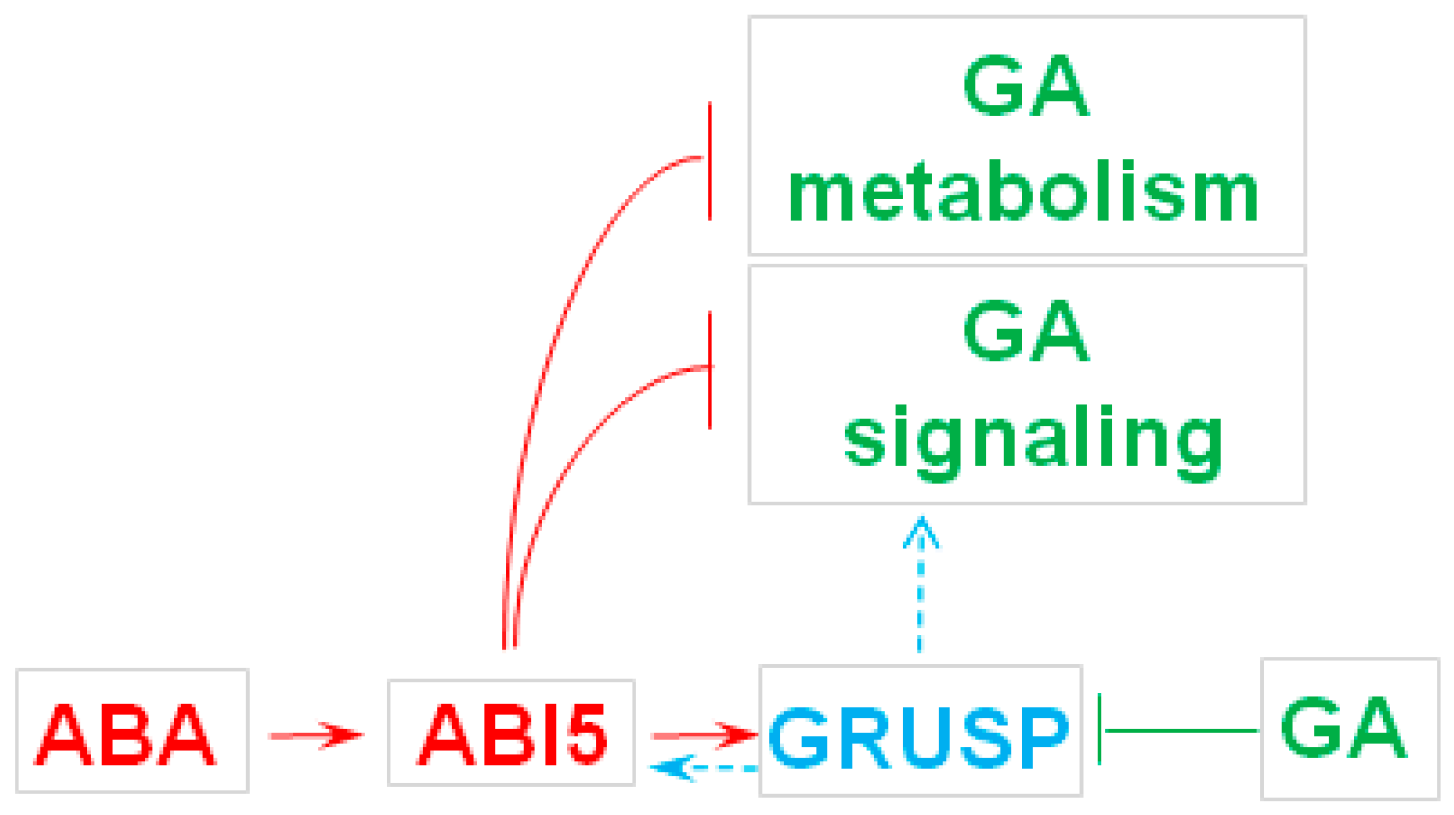
Proposed mechanism through which GRUSP functions in the regulation of ABA- and GA-dependent control of germination. In dry Col-0 seeds, the *GRUSP* transcript level is upregulated, and its expression correlates with elevated level of ABA and enhanced expression of genes involved in ABA signaling and metabolism (marked with red). Suppression of the ABA-dependent response and perception of GA-dependent signals (marked with green) are required for initiation of germination, which is impaired in the *grusp-115* mutant. This indicates that GRUSP (marked with blue) acts as a positive regulator of germination and simultaneously as a negative regulator of ABA signaling during germination, possibly through ABI5 or their associated bZIP genes. GRUSP also regulates GA metabolism directly or by suppressing ABA signaling. Notes: solid line indicate direct regulation and dash line possibly direct or indirect influence.

Rather than indicating that *GRUSP* is a downstream target, these findings demonstrate that GRUSP feedback-represses the ABI5. Combined with the presence of ABI5-binding motifs in the *GRUSP* promoter, these reciprocal profiles strongly suggest a tightly controlled feedback loop where ABI 5 protein stimulates *GRUSP* transcription under stress or dormancy, while the accumulated GRUSP protein subsequently acts to suppress *ABI4/5* expression to facilitate germination initiation (Figure 11).

In summary, our findings demonstrate that GRUSP involved in processes modulating ABA signalling and indirectly affects gibberellin metabolic pathways during early development. Impaired GRUSP activity triggers elevated ABA biosynthesis in dry seeds, which subsequently prevents the timely downregulation of core ABA signalling transcripts—specifically *ABI2*, *ABI3*, *ABI4*, and *ABI5*—upon imbibition. Such a prolonged signalling state significantly retards germination and delays seedling establishment, especially in the presence of ABA. Aligning with established cistromic and genetic frameworks, our data support a model wherein GRUSP acts as a negative regulator of ABA signal transduction during germination by suppressing ABI5, or associated bZIP transcription factors. Given the antagonistic relationship between these phytohormones, GRUSP indirectly coordinates GA metabolism by relieving ABA-mediated transcriptional repression (Figure 11). Furthermore, based on these data and the data presented in this study, we hypothesize that the GA-dependent pathway in *grusp-115* is not directly disrupted but altered secondarily due to higher activation of ABA responses.

## 4. Materials and Methods

### 4.1. Plant Material and Growth Conditions

*Arabidopsis thaliana* (L.) Heynh ecotype Columbia wild type (*Arabidopsis*; Col-0) and the GABI-Kat T-DNA [68] insertion alleles of the *At3g58450* gene, GK_115C08 (NASC N410976), GK_562A03 (NASC 453859), and GK_756D02 (NASC N472518), in the Col-0 background, named *grusp-115*, *grusp-562*, and *grusp-756*, were obtained from the Nottingham Arabidopsis Stock Centre (Nottingham, UK). All the plants were grown side by side in a climate-controlled chamber under standard conditions (16 h light regime, light intensity of 100 μmol m^−2^ s^−1^ photosynthetic flux, 23 °C, 60% relative humidity). For all the assays, the same batches of Col-0 and mutant line seeds were taken. Seeds were surface-sterilized for 10 min in 50% (*v*/*v*) bleach supplemented with 0.02% Tween-20 and then were washed four times with sterile distilled water before being plated on half-strength Murashige–Skoog (Duchefa Biochemie, Haarlem, The Netherlands) medium supplemented with 0.8% phytoagar (Duchefa Biochemie, Haarlem, The Netherlands). Afterward, the seeds were stratified at 4 °C for 3 d in the dark to break dormancy and were transferred to a climate-controlled chamber for germination.

### 4.2. Screening the GRUSP T-DNA Alleles

The T-DNA insertion lines were selected on solid MS medium supplemented with 3% sucrose, 0.8% agar (pH 5.8), and 4.5 μg mL^−1^ sulfadiazine. Resistant plants were subsequently transferred to soil, and individual plants were screened for homozygosity of the T-DNA insertion (Figure S2) by PCR using primers specific to the T-DNA insert (BP; 8474_td Gabi-kat primer) and *At3g58450* (*GRUSP*) gene-specific forward primers (LP1 for *grusp-115*; LP2 for *grusp*-562 and LP3 for *grusp*-756). The primer pair LP1/RP1 was used for genotyping the *grusp-115* mutant, LP2/RP2 for *grusp*-562 and LP3/RP3 for *grusp*-756 (Figure S2A). The transcript levels of the *At3g58450* gene were analyzed in dry seeds of the mutant lines compared with Col-0 by reverse-transcription PCR (RT-PCR) using two pairs of primers, P1/P2, located downstream of all T-DNA inserts (Figure S2C).

All oligonucleotide sequences are listed in Table S1. The extraction of genomic DNA from *Arabidopsis* plants and PCR analysis were performed according to standard procedures by Sambrook et al. (1998) [69]. RNA extraction from dry seeds and RT-PCR are described below.

### 4.3. ABA Germination Response Assays

#### 4.3.1. Seed Germination Assays

For the seed germination and sensitivity assays, the seed batches of Col-0 and the *grusp* mutant lines were harvested simultaneously from dry siliques and stored for 3 months at 20 °C in the dark before analysis. Afterward, the seeds were sown on solid half-strength MS medium supplemented with a range of concentrations of ABA (0.5, 1, and 3 µM; Serva, Heidelberg, Germany) or MS medium without hormone (0 µM) as a control. After seed stratification, as described above, the plates were incubated in a climate-controlled chamber under standard conditions. Germination was scored daily by radicle emergence for up to 7–10 days. Each assay was repeated five times, with three biological replicates containing at least 100 seeds per plate for each genotype. The germination frequency (%) was calculated at 24 h and monitored daily.

An after-ripening assay was performed using seeds of Col-0 and *grusp-115* stored for 1 month or 1 year at 20 °C in the dark.

#### 4.3.2. ABA Inhibition Assay

An ABA inhibition assay was performed as described by Zhao et al. (2014) [70] with minor modifications according to Chiriotto et al. (2023) [49]. Sterile seeds were imbibed for 24 h in half-strength MS medium supplemented with 100 μM fluridone (F) at 4 °C. Afterward, the seeds were washed five times with sterile distilled water and transferred to solid 0.5× MS plates supplemented with 1 μM ABA (ABA+F) or without hormone (MS+F). Control seeds were germinated under the same conditions without prior fluridone treatment (MS or ABA). Germination was scored as described above.

#### 4.3.3. Time-Course Diagram of Developmental Stages

A growth stage timeline of Col-0 and the *grusp* mutant lines during a 10-day growth period was constructed according to the growth stage-based phenotypic analysis described by Boyes et al. [47].

#### 4.3.4. Root Length Analysis

Root growth and ABA sensitivity were evaluated using the method described by Chen et al. (2008) [71].

To investigate the effects of exogenous ABA (0.1 μM; 0.25 μM; 0.5 μM; 0.75 μM, and 3 μM) on root elongation, 3-month after-ripened sterilized seeds of each genotype were germinated directly under the selected conditions or pre-germinated on solid half-strength MS medium in vertically oriented Petri dishes. Standard 90-mm Petri dishes were used for seed planting and kept vertical upon transfer to standard growth conditions. To analyze root growth during initial germination without transplantation, primary root length was measured 10 days after the plants were transferred to light. To study the post-germination effect of ABA, Col-0 and *grusp-115* seedlings reaching 0.5 cm in size (4 days old) were aseptically transplanted into square Petri dishes (120 × 120 mm) containing 1% MS agar medium either without additives (control) or supplemented with 0.1 μM to 3 μM ABA. Root length was recorded 6 days after transplantation. Each data point represents the mean ± SE of three independent biological experiments, with 25 plants analyzed per treatment.

#### 4.3.5. ABA Content Measurement

The ABA content was determined in 3-month-old seeds using an immunoassay method [72]. All procedures were conducted in accordance with the manufacturer’s protocol for the abscisic acid ELISA quantification kit (AS20 4392; Agrisera, Vännäs, Sweden). The seeds were imbibed on Whatman™ 3MM chromatography paper (100 Results Way, Marlborough, MA, USA; medium thickness: 0.34 mm) placed in 35 mm Petri dishes. Approximately 0.5 g of freeze-dried, crushed material obtained from dry seeds or seeds imbibed in 0.5× MS for 24 h at 23 °C was solubilized in 4.5 mL of extraction buffer. The extracts were incubated overnight in the dark at 4 °C and then centrifuged at 15,000 *g* for 15 min, after which the supernatants were subjected to ELISA according to the kit manual (Agrisera). Optical density was measured at 450 nm using a Multiskan MS LabSystems microplate reader (Thermo/LabSystems, Pittsburgh, PA, USA).

### 4.4. RNA Extraction and Real-Time Quantitative PCR (qRT-PCR)

Total RNA was extracted from 100 mg of 7-day-old seedlings and green siliques using TRIzol reagent according to standard instructions. Total RNA from 100 mg of 3-month-old dry and 24 h imbibed seeds was extracted as described previously [73]. Residual genomic DNA contamination was removed by DNAse I (Thermo Fisher Scientific, Waltham, MA, USA) treatment. First-strand cDNA was synthesized using RevertAid Reverse Transcriptase (Thermo Fisher Scientific, Waltham, MA, USA) and a mixture of oligo-dT_18_ primers (Evrogen, Moscow, Russia) with 1 µg of total RNA. qRT-PCR analysis was performed using the qPCRmix-HS SYBR kit (Evrogen, Moscow, Russia) on a ANK-32 real-time PCR system (Syntol, Moscow, Russia). Gene expression was normalized to that of the ubiquitin-conjugating enzyme UBC10 (*At5g53300*) as a highly expressed reference and protein phosphatase 2A (PP2A) regulatory subunit A2 (*At3g25800*) as a lowly expressed reference, according to Pfaffl (2001) [74]. The use of these reference genes was validated for the expression levels of the target genes. The relative expression level was normalized relative to that of the reference genes. The primers used for the qRT-PCR assay are listed in Table S1. Each sample was assayed in triplicate using at least three independent biological replicates.

To analyze *GRUSP* gene expression in response to phytohormones, 7-day-old seedlings grown under standard conditions were transferred for 3 h to Petri dishes containing 0.5× MS-saturated filter paper supplemented with phytohormones. Rosette leaves of 3-, 5-, and 7-week-old Col-0 plants grown on soil in a climate chamber under a 16 h light/8 h dark photoperiod at 23 °C with 60 μmol m^−2^ s^−1^ (control) were sprayed with phytohormones and kept for 3 h, followed by rapid freezing in liquid nitrogen and storage at −70 °C.

Working solutions of phytohormones (Sigma-Aldrich, St. Louis, MO, USA) were used at final concentrations of 5 × 10^−5^ M ABA, 10^−6^ M indole-3-acetic acid (IAA), 5 × 10^−6^ M GA_4+7_, 5 × 10^−6^ M trans-zeatin (tZ), 10^−7^ M brassinolide (BL), 10^−5^ M 1-aminocyclopropane-1-carboxylic acid (ACC), 1 × 10^−5^ M salicylic acid (SA), and 5 × 10^−5^ M methyl jasmonate (MeJA). All concentrations were experimentally determined.

The expression patterns of genes related to ABA and GA metabolism in Col-0 and *grusp-115* seeds were analyzed by qRT-PCR before (dry seeds (DS)) and after 24 h of seed imbibition in 0.5× MS solution without (0; MS) or with 1 µM ABA. Aliquots of 100 mg of seeds were collected, frozen in liquid nitrogen and stored at −80 °C for further analysis. The expression analysis of genes associated with ABA signaling in 7-day-old seedlings was performed by qRT-PCR using samples obtained from seedlings grown on MS medium or in the presence of 1 μM ABA under standard conditions for seven days.

### 4.5. Protein Extraction and Western Blotting

Total protein extract were obtained 10-days-old seedlings as described previously [44]. Total protein extraction from dry and imbibed seeds was performed according to Heidorn-Czarna et al. (2018) [75]. Protein concentration was determined by bicinchoninic acid (BCA) method using Pierce BCA assay kit (Thermo Fisher Scientific, Waltham, MA, USA).

25 µg of total protein samples were mixed with equal volume of 2× sample buffer, denatured for 5 min at 70 °C, separated on 12.5% SDS-PAGE (Next Gel kit, AMRESCO, Solon, OH, USA) and blotted 1 h to PVDF membrane (GE Healthcare, Chicago, IL, USA) using semi-dry transfer cell (Bio-Rad, Hercules, CA, USA). Protein transfer was checked using 0.5% Ponceau S staining. Blots were blocked for 1 h at room temperature (RT) in phosphate buffered saline (PBS) containing 5% non-fat dry milk (Fluka Chemie AG, Buchs, Switzerland) and 0.1% Tween-20, followed by incubation for 2 h at RT with primary antibodies against ABI5 (AS12 1863, Agrisera, Vännäs, Sweden) or Lea 4-5 (loading control; AS13 2758A, Agrisera, Vännäs, Sweden) proteins and diluted to 1: 2000. Immunoreactions were detected using peroxidase-conjugated goat anti-rabbit antibodies (anti-rabbit IgG horse radish peroxidases conjugated, ASO9 602, Agrisera, Vännäs, Sweden). The blots were developed with Clarity Western ECL Substrate (Bio-Rad, Hercules, CA, USA) and signals were obtained using Li-Core image system (Lincoln, NE, USA).

### 4.6. Statistical Analysis

All experiments were performed at least three times. Data were subjected to analysis of variance (one-way ANOVA) followed by post hoc Holm’s multiple-comparison test or Student’s *t* tests using the GraphPad Prism 8.0 statistical software packadge (GraphPad Software, Inc., San Diego, CA, USA) program (accessed on 12 June 2026). The values shown in each figure represent the mean ± standard error (SE).

## 5. Conclusions

In conclusion, this study demonstrates that the functions of the universal stress protein (USP) family extend beyond stress tolerance to pivotal developmental processes, specifically the regulation of seed germination and post-germination growth. The gene *At3g58450*, encoding a putative USP-like protein (GRUSP), is functionally linked to the mechanisms controlling processes that occur in imbibed and germinating seeds of *Arabidopsis thaliana*. Similar to known genes that control germination, *GRUSP* transcript levels were highest in dry seeds but decreased sharply within 24 h of imbibition. Furthermore, *GRUSP* expression is responsive to phytohormonal cues—being induced by ABA and auxin while repressed by GAs—confirming its integration into hormone-regulated germination pathways. Physiological assays established that GRUSP functions during and after germination is tied to ABA networks. Notably, the ABA hypersensitivity exhibited by the *grusp-115* knockout line persisted despite pretreatment with the ABA biosynthesis inhibitor fluridone. This finding indicates that the *grusp-115* phenotype is due not only to elevated endogenous ABA levels *per se*, but from changed hormone perception and signal transduction, as further supported by the disrupted expression profiles of core ABA metabolic and signaling genes. Our data identify GRUSP as a component of negative regulatory mechanisms of ABA signaling during seed imbibition and post-germination growth that indirectly participates in modulation GA metabolic pathways. Consequently, GRUSP represents a novel regulatory protein participating in GA and ABA response during germination, and further elucidation of this molecular interplay remains an area of profound interest.

## Data Availability

The original contributions presented in this study are included in the article/Supplementary Material. Further inquiries can be directed to the corresponding author.

## Supplementary Materials

The following supporting information can be downloaded at: https://www.mdpi.com/article/10.3390/ijms27177540/s1.

## Abbreviations

*Arabidopsis thaliana*	*Arabidopsis*
ABA	Abscisic acid
Col-0	Columbia-0 ecotype; wild type
GA	Gibberellin
GRUSP	Germination-related universal stress protein

## References

[1] Shu K., Liu X.D., Xie Q., He Z.H. (2016). Two Faces of One Seed: Hormonal Regulation of Dormancy and Germination. Mol. Plant.

[2] Kucera B., Cohn M.A., Leubner-Metzger G. (2005). Plant hormone interactions during seed dormancy release and germination. Seed Sci. Res..

[3] Liu X., Hou X. (2018). Antagonistic Regulation of ABA and GA in Metabolism and Signaling Pathways. Front. Plant Sci..

[4] Mitchum M.G., Yamaguchi S., Hanada A., Kuwahara A., Yoshioka Y., Kato T., Tabata S., Kamiya Y., Sun T.P. (2006). Distinct and overlapping roles of two gibberellin 3-oxidases in Arabidopsis development. Plant J..

[5] Rieu I., Ruiz-Rivero O., Fernandez-Garcia N., Griffiths J., Powers S.J., Gong F., Linhartova T., Eriksson S., Nilsson O., Thomas S.G. (2008). The gibberellin biosynthetic genes AtGA20ox1 and AtGA20ox2 act, partially redundantly, to promote growth and development throughout the Arabidopsis life cycle. Plant J..

[6] Rieu I., Eriksson S., Powers S.J., Gong F., Griffiths J., Woolley L., Benlloch R., Nilsson O., Thomas S.G., Hedden P. (2008). Genetic analysis reveals that C19-GA 2-oxidation is a major gibberellin inactivation pathway in Arabidopsis. Plant Cell.

[7] Finkelstein R. (2013). Abscisic Acid synthesis and response. Arab. Book.

[8] Lefebvre V., North H., Frey A., Sotta B., Seo M., Okamoto M., Nambara E., Marion-Poll A. (2006). Functional analysis of Arabidopsis NCED6 and NCED9 genes indicates that ABA synthesized in the endosperm is involved in the induction of seed dormancy. Plant J..

[9] Kushiro T., Okamoto M., Nakabayashi K., Yamagishi K., Kitamura S., Asami T., Hirai N., Koshiba T., Kamiya Y., Nambara E. (2004). The Arabidopsis cytochrome P450 CYP707A encodes ABA 8′-hydroxylases: Key enzymes in ABA catabolism. EMBO J..

[10] Vishal B., Kumar P.P. (2018). Regulation of Seed Germination and Abiotic Stresses by Gibberellins and Abscisic Acid. Front. Plant Sci..

[11] Yano R., Kanno Y., Jikumaru Y., Nakabayashi K., Kamiya Y., Nambara E. (2009). CHOTTO1, a putative double APETALA2 repeat transcription factor, is involved in abscisic acid-mediated repression of gibberellin biosynthesis during seed germination in Arabidopsis. Plant Physiol..

[12] Ko J.H., Yang S.H., Han K.H. (2006). Upregulation of an Arabidopsis RING-H2 gene, XERICO, confers drought tolerance through increased abscisic acid biosynthesis. Plant J..

[13] Zentella R., Zhang Z.L., Park M., Thomas S.G., Endo A., Murase K., Fleet C.M., Jikumaru Y., Nambara E., Kamiya Y. (2007). Global analysis of della direct targets in early gibberellin signaling in Arabidopsis. Plant Cell.

[14] Dill A., Thomas S.G., Hu J., Steber C.M., Sun T.P. (2004). The Arabidopsis F-box protein SLEEPY1 targets gibberellin signaling repressors for gibberellin-induced degradation. Plant Cell.

[15] Hauvermale A.L., Ariizumi T., Steber C.M. (2012). Gibberellin signaling: A theme and variations on DELLA repression. Plant Physiol..

[16] Lopez-Molina L., Mongrand S., Chua N.H. (2001). A postgermination developmental arrest checkpoint is mediated by abscisic acid and requires the ABI5 transcription factor in Arabidopsis. Proc. Natl. Acad. Sci. USA.

[17] Park S.Y., Fung P., Nishimura N., Jensen D.R., Fujii H., Zhao Y., Lumba S., Santiago J., Rodrigues A., Chow T.F. (2009). Abscisic acid inhibits type 2C protein phosphatases via the PYR/PYL family of START proteins. Science.

[18] Miyakawa T., Fujita Y., Yamaguchi-Shinozaki K., Tanokura M. (2013). Structure and function of abscisic acid receptors. Trends Plant Sci..

[19] Piskurewicz U., Jikumaru Y., Kinoshita N., Nambara E., Kamiya Y., Lopez-Molina L. (2008). The gibberellic acid signaling repressor RGL2 inhibits Arabidopsis seed germination by stimulating abscisic acid synthesis and ABI5 activity. Plant Cell.

[20] Shu K., Zhang H., Wang S., Chen M., Wu Y., Tang S., Liu C., Feng Y., Cao X., Xie Q. (2013). ABI4 regulates primary seed dormancy by regulating the biogenesis of abscisic acid and gibberellins in arabidopsis. PLoS Genet..

[21] Davière J.M., Achard P.A. (2016). Pivotal Role of DELLAs in Regulating Multiple Hormone Signals. Mol. Plant.

[22] Skubacz A., Daszkowska-Golec A., Szarejko I. (2016). The Role and Regulation of ABI5 (ABA-Insensitive 5) in Plant Development, Abiotic Stress Responses and Phytohormone Crosstalk. Front. Plant Sci..

[23] Ogawa M., Hanada A., Yamauchi Y., Kuwahara A., Kamiya Y., Yamaguchi S. (2003). Gibberellin biosynthesis and response during Arabidopsis seed germination. Plant Cell.

[24] Nakabayashi K., Okamoto M., Koshiba T., Kamiya Y., Nambara E. (2005). Genome-wide profiling of stored mRNA in Arabidopsis thaliana seed germination: Epigenetic and genetic regulation of transcription in seed. Plant J..

[25] Cao D., Cheng H., Wu W., Soo H.M., Peng J. (2006). Gibberellin mobilizes distinct DELLA-dependent transcriptomes to regulate seed germination and floral development in Arabidopsis. Plant Physiol..

[26] Reeves W.M., Lynch T.J., Mobin R., Finkelstein R.R. (2011). Direct targets of the transcription factors ABA-Insensitive(ABI)4 and ABI5 reveal synergistic action by ABI4 and several bZIP ABA response factors. Plant Mol. Biol..

[27] Nyström T., Neidhardt F.C. (1992). Cloning, mapping and nucleotide sequencing of a gene encoding a universal stress protein in Escherichia coli. Mol. Microbiol..

[28] Zarembinski T.I., Hung L.W., Mueller-Dieckmann H.J., Kim K.K., Yokota H., Kim R., Kim S.H. (1998). Structure-based assignment of the biochemical function of a hypothetical protein: A test case of structural genomics. Proc. Natl. Acad. Sci. USA.

[29] Tkaczuk K.L., A Shumilin I., Chruszcz M., Evdokimova E., Savchenko A., Minor W. (2013). Structural and functional insight into the universal stress protein family. Evol. Appl..

[30] Heermann R., Jung K. (2010). The complexity of the ‘simple’ two-component system KdpD/KdpE in Escherichia coli. FEMS Microbiol. Lett..

[31] Chi Y.H., Koo S.S., Oh H.T., Lee E.S., Park J.H., Phan K.A.T., Wi S.D., Bae S.B., Paeng S.K., Chae H.B. (2019). The Physiological Functions of Universal Stress Proteins and Their Molecular Mechanism to Protect Plants from Environmental Stresses. Front. Plant Sci..

[32] Kerk D., Bulgrien J., Smith D.W., Gribskov M. (2003). Arabidopsis proteins containing similarity to the universal stress protein domain of bacteria. Plant Physiol..

[33] Loukehaich R., Wang T., Ouyang B., Ziaf K., Li H., Zhang J., Lu Y., Ye Z. (2012). SpUSP, an annexin-interacting universal stress protein, enhances drought tolerance in tomato. J. Exp. Bot..

[34] Udawat P., Jha R.K., Sinha D., Mishra A., Jha B. (2016). Overexpression of a Cytosolic Abiotic Stress Responsive Universal Stress Protein (SbUSP) Mitigates Salt and Osmotic Stress in Transgenic Tobacco Plants. Front. Plant Sci..

[35] Jung Y.J., Melencion S.M.B., Lee E.S., Park J.H., Alinapon C.V., Oh H.T., Yun D.-J., Chi Y.H., Lee S.Y. (2015). Universal stress protein exhibits a redox-dependent chaperone function in *Arabidopsis* and enhances plant tolerance to heat shock and oxidative stress. Front. Plant Sci..

[36] Luo D., Wu Z., Bai Q., Zhang Y., Huang M., Huang Y., Li X. (2023). Universal Stress Proteins: From Gene to Function. Int. J. Mol. Sci..

[37] Bhuria M., Goel P., Kumar S., Singh A.K. (2016). The Promoter of AtUSP Is Co-regulated by Phytohormones and Abiotic Stresses in Arabidopsis thaliana. Front. Plant Sci..

[38] Gorshkova D.S., Pojidaeva E.S. (2021). Effect of Phytohormones on the Expression of USP Genes in Arabidopsis thaliana Seedlings. Mosc. Univ. Biol. Sci. Bull..

[39] Sauter M., Rzewuski G., Marwedel T., Lorbiecke R. (2002). The novel ethylene-regulated gene OsUsp1 from rice encodes a member of a plant protein family related to prokaryotic universal stress proteins. J. Exp. Bot..

[40] Müssig C., Shin G.H., Altmann T. (2003). Brassinosteroids promote root growth in Arabidopsis. Plant Physiol..

[41] Zik M., Irish V.F. (2003). Global identification of target genes regulated by APETALA3 and PISTILLATA floral homeotic gene action. Plant Cell.

[42] Lee L.Y., Hou X., Fang L., Fan S., Kumar P.P., Yu H. (2012). STUNTED mediates the control of cell proliferation by GA in Arabidopsis. Development.

[43] Gorshkova D.S., Getman I.A., Voronkov A.S., Chizhiva S.I., Kuznetsov V.V., Pojidaeva E.S. (2018). The Gene Encoding the Universal Stress Protein AtUSP is Regulated by Phytohormones and Involved in Seed Germination of *Arabidopsis thaliana*. Dokl. Biochem. Biophys..

[44] Gorshkova D.S., Pojidaeva E.S. (2021). Members of the Universal Stress Protein Family are Indirectly Involved in Gibberellin-Dependent Regulation of Germination and Post-Germination Growth. Russ. J. Plant Physiol..

[45] Gorshkova D.S., Getman I.A., Sergeeva L.I., Kuznetsov V.V., Pojidaeva E.S. (2021). GRUSP, an Universal Stress Protein, Is Involved in Gibberellin-dependent Induction of Flowering in *Arabidopsis thaliana*. Dokl. Biochem. Biophys..

[46] Yoshida T., Fujita Y., Sayama H., Kidokoro S., Maruyama K., Mizoi J., Shinozaki K., Yamaguchi-Shinozak K. (2010). AREB1, AREB2, and ABF3 are master transcription factors that cooperatively regulate ABRE-dependent ABA signaling involved in drought stress tolerance and require ABA for full activation. Plant J..

[47] Boyes D.C., Zayed A.M., Ascenzi R., McCaskill A.J., Hoffman N.E., Davis K.R., Görlach J. (2001). Growth stage-based phenotypic analysis of Arabidopsis: A model for high throughput functional genomics in plants. Plant Cell.

[48] Bartels P.G., Watson C.W. (1978). Inhibition of Carotenoid Synthesis by Fluridone and Norflurazon. Weed Sci..

[49] Chiriotto T.S., Saura-Sánchez M., Barraza C., Botto J.F. (2023). BBX24 Increases Saline and Osmotic Tolerance through ABA Signaling in Arabidopsis Seeds. Plants.

[50] Bentsink L., Jowett J., Hanhart C.J., Koornneef M. (2006). Cloning of DOG1, a quantitative trait locus controlling seed dormancy in *Arabidopsis*. Proc. Natl. Acad. Sci. USA.

[51] Zhao H., Zhang Y., Zheng Y. (2022). Integration of ABA, GA, and light signaling in seed germination through the regulation of ABI5. Front. Plant Sci..

[52] Wang Y., Li L., Ye T., Lu Y., Chen X., Wu Y. (2013). The inhibitory effect of ABA on floral transition is mediated by ABI5 in Arabidopsis. J. Exp. Bot..

[53] Chiu R.S., Nahal H., Provart N.J., Gazzarrini S. (2012). The role of the Arabidopsis FUSCA3 transcription factor during inhibition of seed germination at high temperature. BMC Plant Biol..

[54] Matilla A.J. (2020). Auxin: Hormonal Signal Required for Seed Development and Dormancy. Plants.

[55] Li X., Chen L., Forde B.G., Davies W.J. (2017). The Biphasic Root Growth Response to Abscisic Acid in Arabidopsis Involves Interaction with Ethylene and Auxin Signalling Pathways. Front. Plant Sci..

[56] Miao R., Yuan W., Wang Y., Garcia-Maquilon I., Dang X., Li Y., Zhang J., Zhu Y., Rodriguez P.L., Xu W. (2021). Low ABA concentration promotes root growth and hydrotropism through relief of ABA INSENSITIVE 1-mediated inhibition of plasma membrane H^+^-ATPase 2. Sci. Adv..

[57] Sun L.R., Wang Y.B., He S.B., Hao F.S. (2018). Mechanisms for Abscisic Acid Inhibition of Primary Root Growth. Plant Signal. Behav..

[58] Luo X., Xu J., Zheng C., Yang Y., Wang L., Zhang R., Ren X., Wei S., Aziz U., Du J. (2023). Abscisic acid inhibits primary root growth by impairing ABI4-mediated cell cycle and auxin biosynthesis. Plant Physiol..

[59] Ali-Rachedi S., Bouinot D., Wagner M.H., Bonnet M., Sotta B., Grappin P., Jullien M. (2004). Changes in endogenous abscisic acid levels during dormancy release and maintenance of mature seeds: Studies with the Cape Verde Islands ecotype, the dormant model of *Arabidopsis thaliana*. Planta.

[60] Huo H., Wei S., Bradford K.J. (2016). DELAY OF GERMINATION1 (DOG1) regulates both seed dormancy and flowering time through microRNA pathways. Proc. Natl. Acad. Sci. USA.

[61] Xi W., Liu C., Hou X., Yu H. (2010). MOTHER OF FT AND TFL1 regulates seed germination through a negative feedback loop modulating ABA signaling in Arabidopsis. Plant Cell.

[62] Yu L.H., Wu J., Zhang Z.S., Miao Z.Q., Zhao P.X., Wang Z., Xiang C.B. (2017). Arabidopsis MADS-Box Transcription Factor AGL21 Acts as Environmental Surveillance of Seed Germination by Regulating ABI5 Expression. Mol. Plant.

[63] Yang C., Li X., Chen S., Liu C., Yang L., Li K., Liao J., Zheng X., Li H., Li Y. (2023). ABI5-FLZ13 module transcriptionally represses growth-related genes to delay seed germination in response to ABA. Plant Commun..

[64] Brocard I.M., Lynch T.J., Finkelstein R.R. (2002). Regulation and Role of the Arabidopsis Abscisic Acid-Insensitive 5 Gene in Abscisic Acid, Sugar, and Stress Response. Plant Physiol..

[65] Bachmair A., Finley D., Varshavsky A. (1986). In vivo half-life of a protein is a function of its amino-terminal residue. Science.

[66] Peng J., Carol P., Richards D.E., King K.E., Cowling R.J., Murphy G.P., Harberd N.P. (1997). The Arabidopsis GAI gene defines a signaling pathway that negatively regulates gibberellin responses. Genes Dev..

[67] Lim S., Park J., Lee N., Jeong J., Toh S., Watanabe A., Kim J., Kang H., Kim D.H., Kawakami N. (2013). ABA-insensitive3, ABA-insensitive5, and DELLAs Interact to activate the expression of SOMNUS and other high-temperature-inducible genes in imbibed seeds in Arabidopsis. Plant Cell.

[68] Kleinboelting N., Huep G., Kloetgen A., Viehoever P., Weisshaar B. (2012). GABI-Kat Simple Search: New features of the *Arabidopsis thaliana* T-DNA mutant database. Nucleic Acids Res..

[69] Sambrook J., Fritsch E.F., Maniatis T. (1989). Molecular Cloning: A Laboratory Manual.

[70] Zhao H., Zhang H., Cui P., Ding F., Wang G., Li R., Jenks M.A., Lü S., Xiong L. (2014). The putative E3 ubiquitin ligase ECERIFERUM9 regulates abscisic acid biosynthesis and response during seed germination and postgermination growth in Arabidopsis. Plant Physiol..

[71] Chen H., Zhang J., Neff M.M., Hong S.-W., Zhang H., Deng X.-W., Xiong L. (2008). Integration of light and abscisic acid signaling during seed germination and early seedling development. Proc. Natl. Acad. Sci. USA.

[72] Liu N., Ding Y., Fromm M., Avramova Z. (2014). Endogenous ABA Extraction and Measurement from *Arabidopsis* Leaves. Bio Protoc..

[73] Meng L., Feldman L. (2010). A rapid TRIzol-based two-step method for DNA-free RNA extraction from Arabidopsis siliques and dry seeds. Biotechnol. J..

[74] Pfaffl M.W. (2001). A new mathematical model for relative quantification in real-time RT-PCR. Nucleic Acids Res..

[75] Heidorn-Czarna M., Domanski D., Kwasniak-Owczarek M., Janska H. (2018). Targeted Proteomics Approach Toward Understanding the Role of the Mitochondrial Protease FTSH4 in the Biogenesis of OXPHOS During Arabidopsis Seed Germination. Front Plant Sci..

