# Supplementary material for: Germination-Related Universal Stress Protein (GRUSP) Is Involved in Abscisic Acid Responses During Imbibition and Early Post-Germination Growth of Arabidopsis Seeds
Source: Int J Mol Sci. 2026 Aug 23;27(17):7540. doi: 10.3390/ijms27177540 (PMC13566284; doi:10.3390/ijms27177540)
Supplement: Supplementary file 1 [file ijms-27-07540-s001.zip › ijms-4437445-supplementary.pdf]

Supplementary Materials

Germination-Related Universal Stress Protein (GRUSP) Is Involved in Abscisic Acid Responses During Imbibition and Early Post-Germination Growth of *Arabidopsis* Seeds

Elena S. Pojidaeva*, Darya S. Gorshkova, Natalia V. Kudryakova, and Victor V. Kusnetsov

K.A. Timiryazev Institute of Plant Physiology RAS, 35 Botanicheskaya St., Moscow 127276, Russia; nvkudryakova@mail.ru (N.V.K.); vkusnetsov2001@mail.ru (V.V.K.)

*Correspondence: alenapoj@mail.ru; Tel.: +7-(499)-678-54-25

Table S1. List of primers used in this research study.

No.	Primer name	Sequence (5'->3')	AGI gene code *Arabidopsis*	Notes
For analysis of lines GK_115C08 (grusp-115), GK_562A03 (grusp-562), and GK_756D02 (grusp-756)				
**1**	**BP; (8474_td)**	ATAATAACGCTGCGGACATCTACATTTT	-	Gabi-kat primer for T-DNA insertion [60]
**2**	**LP1**	CAATGAGGAAAGCACGAGAAG	*At3g58450*	*grusp-115* genotyping
**3**	**RP1**	CTCACCGTAGCAGTGGAGATC		
**4**	**LP2**	TCCATATTTCACAGACCCGAG	*At3g58450*	*grusp-562* genotyping
**5**	**RP2**	CTTCTCGTGCTTTCCTCATTG		
**6**	**LP3**	AACCGAATTTAAATCGGTTCG	*At3g58450*	*grusp-756* genotyping
**7**	**RP3**	GTACCTTTTGATCATGCCGAG		
**8**	**P1**	CGACCGCCTAGAGAAACCTC	*At3g58450*	*GRUSP* expression
**9**	**P2**	GAACTTTACTTTGCAAAAGTAAAC		
For reverse-transcription PCR (RT-PCR) and quantitative reverse-transcription PCR (qRT-PCR)				
Reference genes				
**10**	**PP2A_F**	ACTGCATCTAAAGACAGAGTTCCA	*At3g25800*	Subunit of protein phosphatase 2A
**11**	**PP2A_R**	CCACAAGCCCAGGACGAAT		
**12**	**UBQ10_F**	CACCATTTCCAAGGTGCTGC	*At5g53300*	Ubiquitin-conjugating enzyme 10
**13**	**UBQ10_R**	GAGCTATCTCGGGCACCAAA		
**ABA metabolism and signaling**				
**14**	**ABI1_F**	CGGCAAAACTGCACTTCCATT	*At4g26080*	*ABI1*
**14**	**ABI1_R**	CACGAGCTCCATTCCACTGAA		
**16**	**ABI2_F**	CTCGCAATGTCAAGATCCATTGGC	*At5g57050*	*ABI2*
**17**	**ABI2_R**	TTACTCGCCGCACTGAAGTCAC		
**18**	**ABI3_F**	GATTGAATCAGCGGCAAGAA	*At3g24650*	*ABI3*
**19**	**ABI3_R**	GTTGTTGTGGTGGTGGAGGA		

**20**	**ABI4_F**	TAGGGCAGGAACAAGGAGGA	*At2g40220*	*ABI4*
**21**	**ABI4_R**	ACGGCGGTGGATGAGTTATT	*At2g40220*	*ABI4*
**22**	**ABI5_F**	TTTGCTATGGAGGTGGCGTT	*At2g36270*	*ABI5*
**23**	**ABI5_R**	TTCCTTCCCCTTAGCCCTC	*At2g36270*	*ABI5*
**24**	**DOG1_F**	GCTCTAATTTGGATGGGTGGT	*At5g45830*	*DOG1*
**25**	**DOG1_R**	GTCGATGTTGCGGAGAAACT	*At5g45830*	*DOG1*
**26**	**CYP1_F**	GGCTCCAAAACCCAATACG	*At4g19230*	*CYP707A1*
**27**	**CYP1_R**	TCCAACAAGTGACCAACTGTA	*At4g19230*	*CYP707A1*
**28**	**CYP2_F**	ATCCTCCAAGCAAAGAGCGT	*At2g29090*	*CYP707A2*
**29**	**CYP2_R**	GGGTGTTGATGGACTTTTGG	*At2g29090*	*CYP707A2*
**30**	**RD29b_F**	GTGAGAGGTGGTGTAACGG	*At5g52300*	*RD29b*
**31**	**RD29b_R**	GTCGGTGCCTCTCTTTTCG	*At5g52300*	*RD29b*
**32**	**ZEP_F**	TAGGTGTTGGGCTTGGTCC	*At5g67030*	*ZEP*
**33**	**ZEP_R**	CAATCGTCGCCGTGTGGAA	*At5g67030*	*ZEP*
**34**	**NCED6_F**	CTTCCTAAACCGACCGACC	*At3g24220*	*NCED6*
**35**	**NCED6_R**	TAACGGCGGAAACATAGGGT	*At3g24220*	*NCED6*
**36**	**NCED9_F**	TAGAAACTTCGGGTCGGTAC	*At1g78390*	*NCED9*
**37**	**NCED9_R**	GTTCTCGGGTTTCGGGATC	*At1g78390*	*NCED9*
**38**	**GA20-1F**	GGCTACGCAAGCAGTTTCAC	*At4g25420*	*GA20ox1*
**39**	**GA20-1R**	TTGAACGGATTTTGAGCGGC	*At4g25420*	*GA20ox1*
**40**	**GA20-3F**	CACTGCGACCCAACATCTCT	*At5g07200*	*GA20ox3*
**41**	**GA20-3R**	CGAAAGCGTGAGGGTTAGGA	*At5g07200*	*GA20ox3*
**42**	**GA3-1F**	AGTCTCTTCGGGCTACCTGT	*At1g15550*	*GA3ox1*
**43**	**GA3-1R**	CGGAAATCGTTGAGAGGCG	*At1g15550*	*GA3ox1*
**44**	**GA2-2F**	GGAACACACAGACCCACAGA	*At1g30040*	*GA2ox2*
**45**	**GA2-2R**	CAGCGACCCAACTTCCATCT	*At1g30040*	*GA2ox2*
**46**	**GA2-6F**	CACTATCCACCAGCACCGTA	*At1g02400*	*GA2ox6*
**47**	**GA2-6R**	AGGGTCAGATGGGATTGGGA	*At1g02400*	*GA2ox6*

Table S2. Endogenous content of bioactive GAs in rosette leaves of Col-0 and *grusp-115*.

**Bioactive GAs**	**Bioactive GA content in rosette leaves, ng/g dry weight**	
	**Col 0**	***grusp*-115**
GA_7	5.158 ± 0.81	6.437 ± 0.56
GA_4	0.239 ± 0.11 [a]	1.222 ± 0.23 [b]
GA_1	1.960 ± 0.21 [a]	0.567 ± 0.11 [b]
GA_3	1.954 ± 0.35 [a]	1.177 ± 0.12 [b]

The content of active GAs was determined in 10 mg of lyophilized samples obtained from rosette leaves of 4-week-old Col-0 and *grusp-115* plants as described in [46]. Values marked with different letters indicate significant differences between the variants based on the *t*-test ($p < 0.05$).

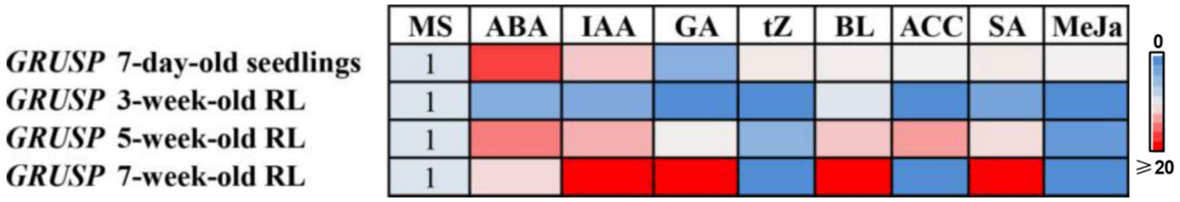

Figure S1. The effects of phytohormones on the expression of the *GRUSP* (*At3g58450*) gene (heat map) in seedlings grown for 7 days on Murashige and Skoog (MS) medium in Petri dishes and in rosette leaves (RL) of 3-, 5-, and 7-week-old Col-0 plants grown on soil in a climate chamber under a 16 h light/8 h dark photoperiod at 23 °C with 60 μmol m^{-2} s^{-1} (control). The "MS" column indicates the baseline ratio of *GRUSP* expression in Col-0 without treatments. Seedling and rosette leaves were exposed for 3 hours to $5 \cdot 10^{-5}$ M ABA, 10^{-6} M indole-3-acetic acid (IAA), $5 \cdot 10^{-6}$ M GA_{4+7}, $5 \cdot 10^{-6}$ M trans-zeatin (tZ), 10^{-7} M brassinolide (BL), 10^{-5} M 1-aminocyclopropane-1-carboxylic acid (ACC), $1 \cdot 10^{-5}$ M salicylic acid (SA), and $5 \cdot 10^{-5}$ M methyl jasmonate (MeJA).

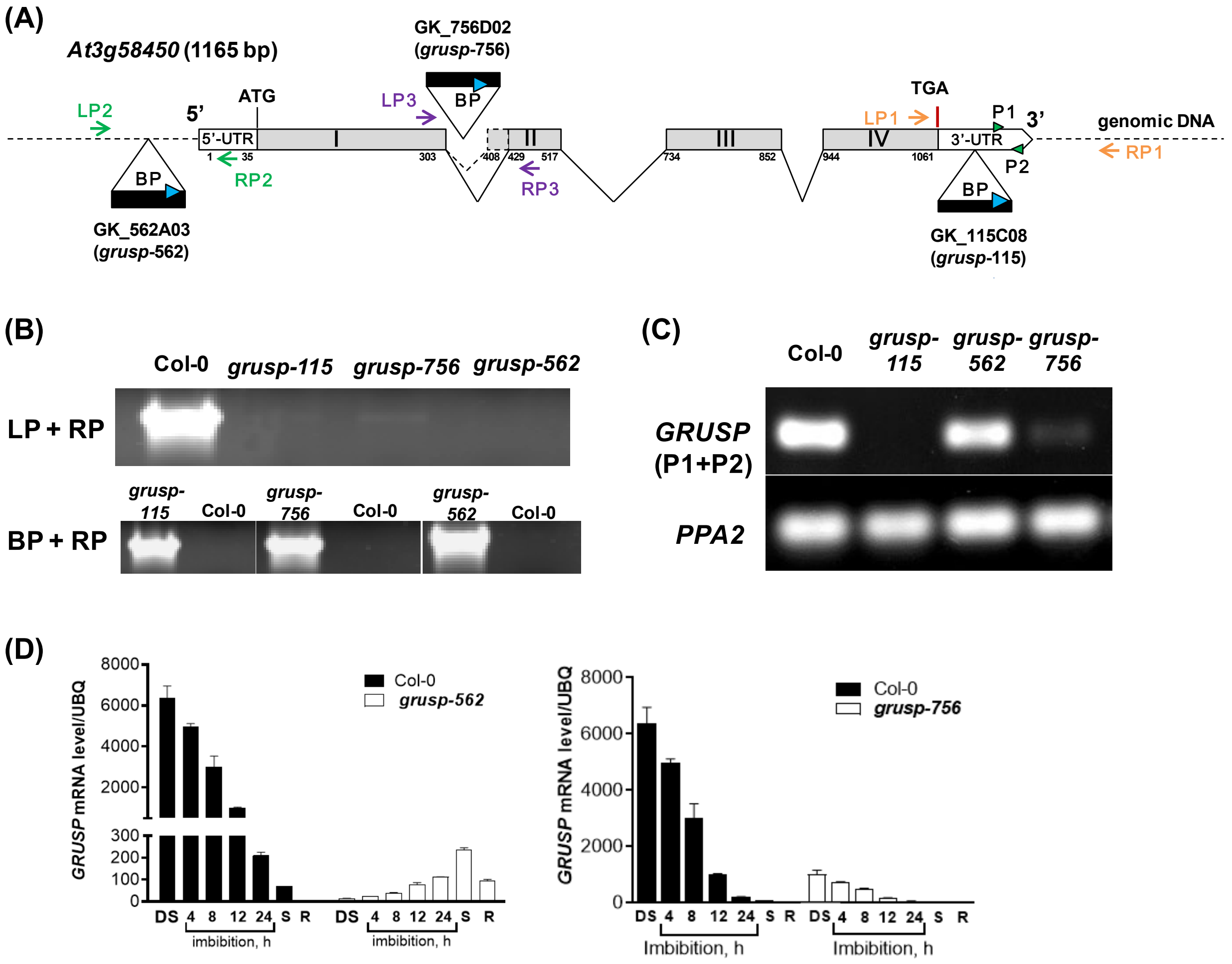

Figure S2. Characterization of *grusp* T-DNA lines.

(**A**) Schematic representation of *Atg358450* gene with indication of T-DNA insertions for GABI-kat *grusp-115, grusp-562,* and *grusp-756* alleles.

(**B**) Results of PCR analysis of genomic DNAs using a primer set of gene-specific (LP/RP) and T-DNA-specific (BP; GABI_kat line (8474_td)) primers. For *grusp-115*, the LP1/RP1 primer pair amplifies a 1065 bp fragment corresponding to the wild type; for *grusp*-562, the LP2/RP2 primer pair amplifies a 1092 bp fragment; for *grusp*-756, the LP3/RP3 primer pair amplifies a 1156 bp fragment; for *grusp*-080638, the LP3/RP3 primer pair amplifies a 1032 bp fragment.

The position of the T-DNA insert was detected using the RP1/BP primers, which amplify, for *grusp-115*, a 480 bp PCR fragment. Accordingly, for *grusp-562*, the primers RP2/BP amplify a 600 bp fragment, and for *grusp-756*, the primers RP3/BP amplify a 500 bp fragment.

(**C**) Relative expression level of *Atg358450* in Col-0 and *grusp* lines obtained by RT-PCR using RNA isolated from green siliques with the primer pair P1/P2 located downstream of T-DNA inserts. The values of the mRNA level are compared to the relative level of *PPA2* gene expression. Primer sequences are listed in Table S1.

(**D**) *GRUSP* transcript levels in Col-0, *grusp-562* and *grusp-756* in dry seeds (DS), after 24 hours of imbibition, in 10-day-old seedlings (S), and in 3-week-old rosette leaves (R). The seeds were imbibed in water for a 24 h time course (4, 8, 12, and 24 h) under a 16 h light/8 h dark photoperiod

at 23 °C with 100 µmol m^{-2} s^{-1}. Zero point (0) corresponds to dry seeds. Relative units were normalized to the reference gene *UBC10*. Primer sequences are listed in Table S1.

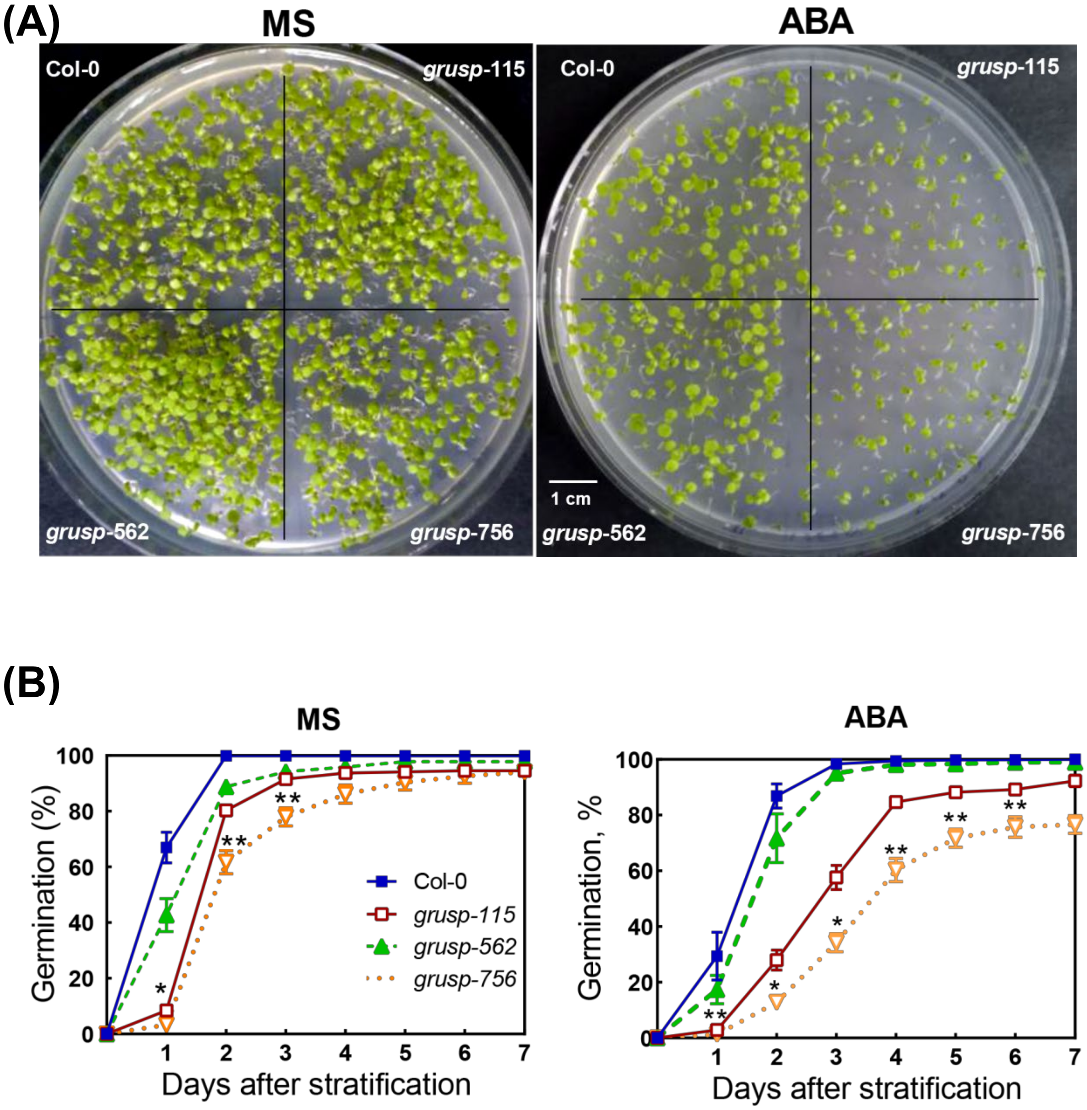

Figure S3. Phenotypes of *grusp* GABI-kat T-DNA insertion alleles versus Col-0.

(**A**) Photographs of 10-day-old Col-0, *grusp-115*, *grusp-562*, and *grusp-756* seedlings grown under standard conditions on solid half-strength MS medium without (MS) or supplemented with 1 µM ABA (ABA). Scale: 1 cm.

(**B**) Germination rates of Col-0, *grusp-115*, *grusp-562*, and *grusp-756* seeds in the presence or absence of 1 µM ABA. Time scale indicates days after vernalization. Radicle emergence was used as a morphological marker for germination. Each data point represents the mean ± SE (*n*=3), with 100 seeds used for each value. Asterisks indicate statistically significant differences between the Col-0 and *grusp-752* genotypes at the * $p < 0.05$ and ** $p < 0.01$ levels (*t*-test).

1 month 1 year

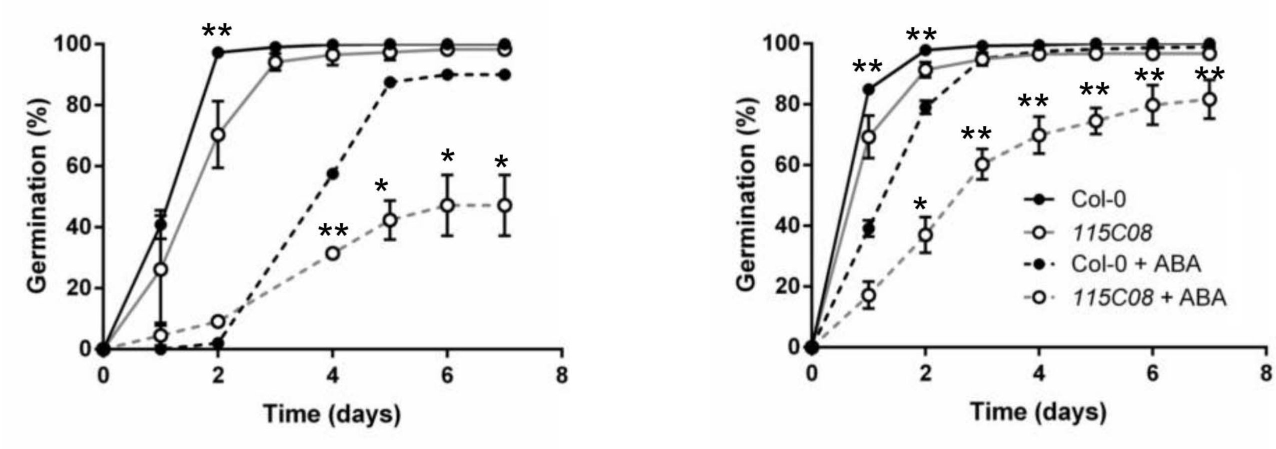

Figure S4. Germination rates of Col-0 and *grusp-115* seeds (*115C08*) versus after-ripening dependence (AR; 1 month and 1 year). Seeds were germinated under standard conditions on solid half-strength MS medium or MS supplemented with 1 μM abscisic acid (ABA). Time scale indicates days after vernalization. Radicle emergence was used as a morphological marker for germination. Each data point represents the mean ± SE (n=3), with 100 seeds used for each value. Asterisks indicate statistically significant differences between ABA-treated seeds of *grusp-115* and Col-0, * $p < 0.05$ and ** $p < 0.01$, as determined by Student's *t*-test. Arrows show the amplitude of differences.

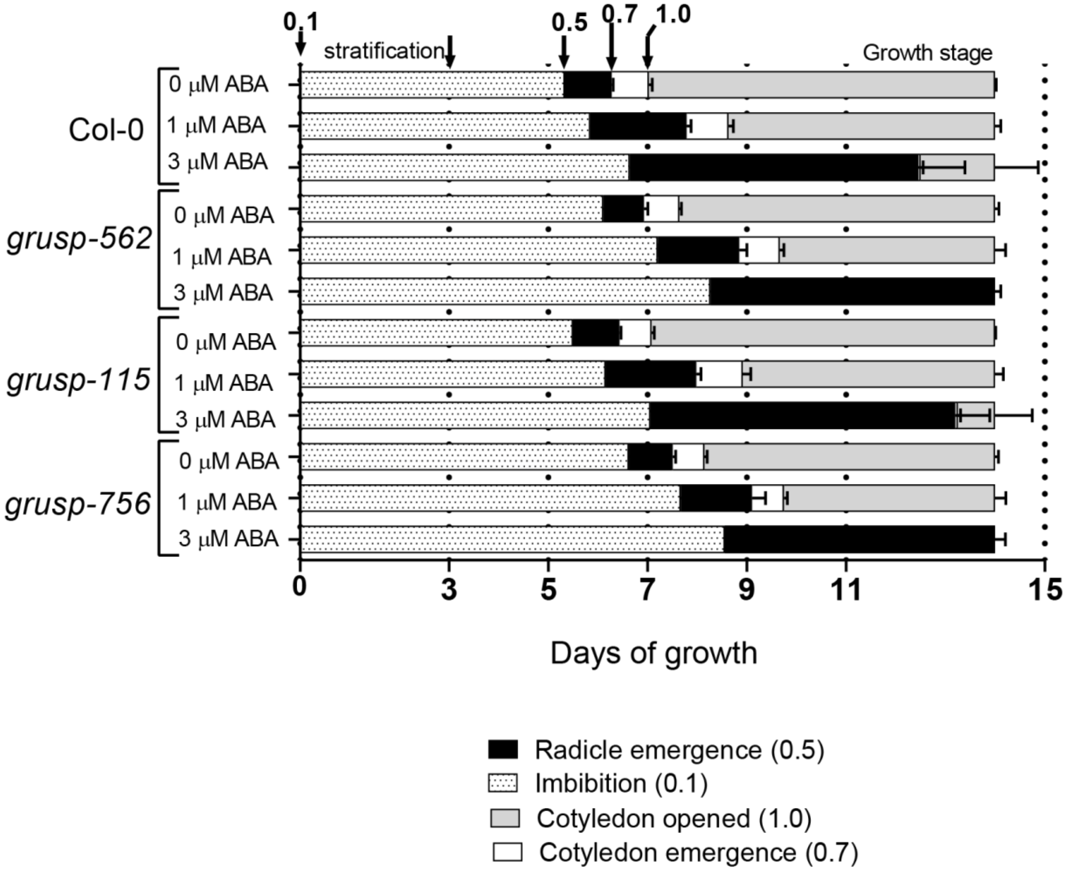

Figure S5. Developmental time-course diagrams of Col-0 and *grusp-115* plants. Seeds of both lines were germinated on solid half-strength Murashige and Skoog (MS) medium (control) supplemented with 1 μM or 3 μM ABA. Starting from stratification (3 days) and transfer of seeds to a growth chamber (day 3), seed counts were performed daily for 10 days; the indicated stages correspond to the standard classification of *Arabidopsis* developmental stages described by Boyes et al. (2001) [47]. The data represent the means ± SE. Each measurement was carried out in three biological replicates of 100 seeds per Petri dish.

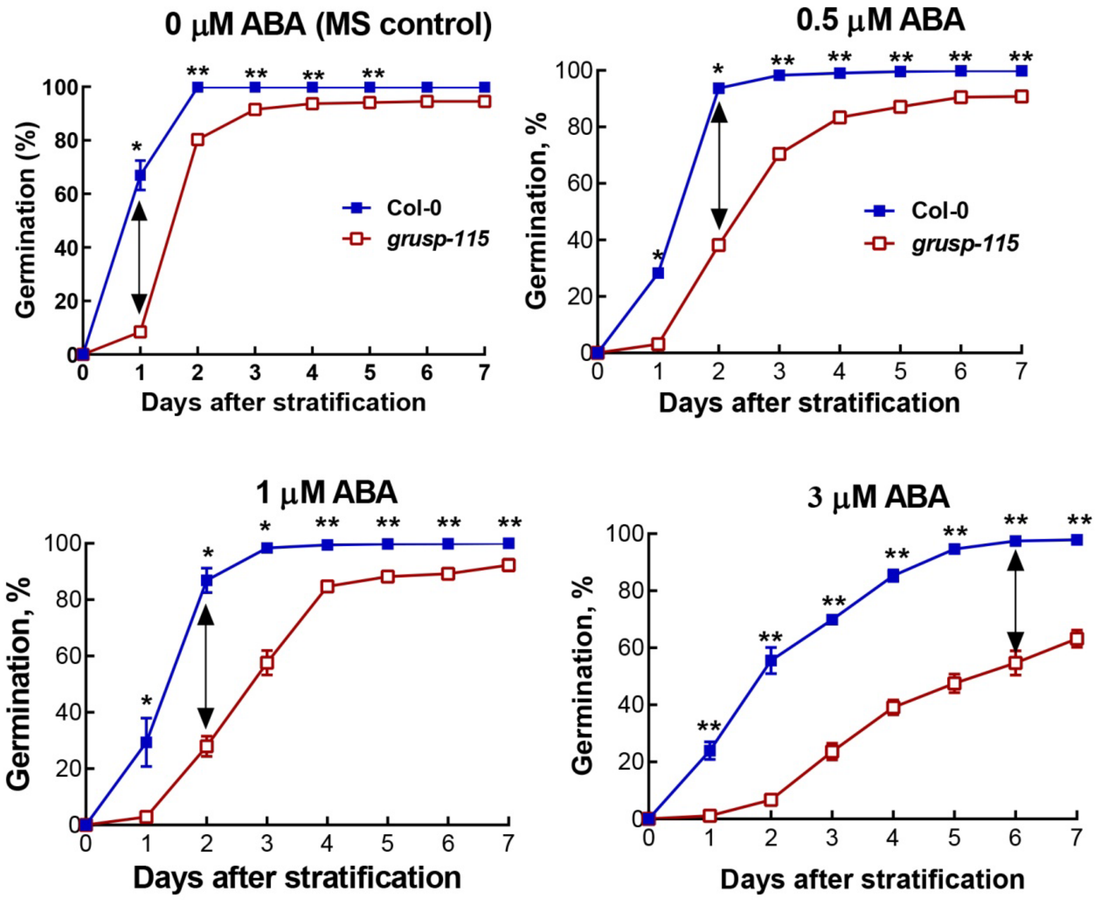

Figure S6. Effects of ABA on germination rate of Col-0 and *grusp-115* seeds. The seeds were germinated on solid half-strength MS plates supplemented with 0.5 μM, 1 μM, 3 μM ABA or without ABA addition (0 μM ABA; MS control) for seven days under standard conditions (16 h day, light intensity 100 μmol m^{-2} s^{-1} photosynthetic flux, 23°C/8 h dark, 60% relative humidity). The time scale indicates days after vernalization. Radicle emergence was used as a morphological marker for germination. Each data point represents the mean ± SE of three replicates, each using 100 seeds. Asterisks indicate statistically significant differences compared with Col-0, * $p < 0.05$ and ** $p < 0.01$, as determined by Student's *t*-test. Arrows show the amplitude of differences.

(A)

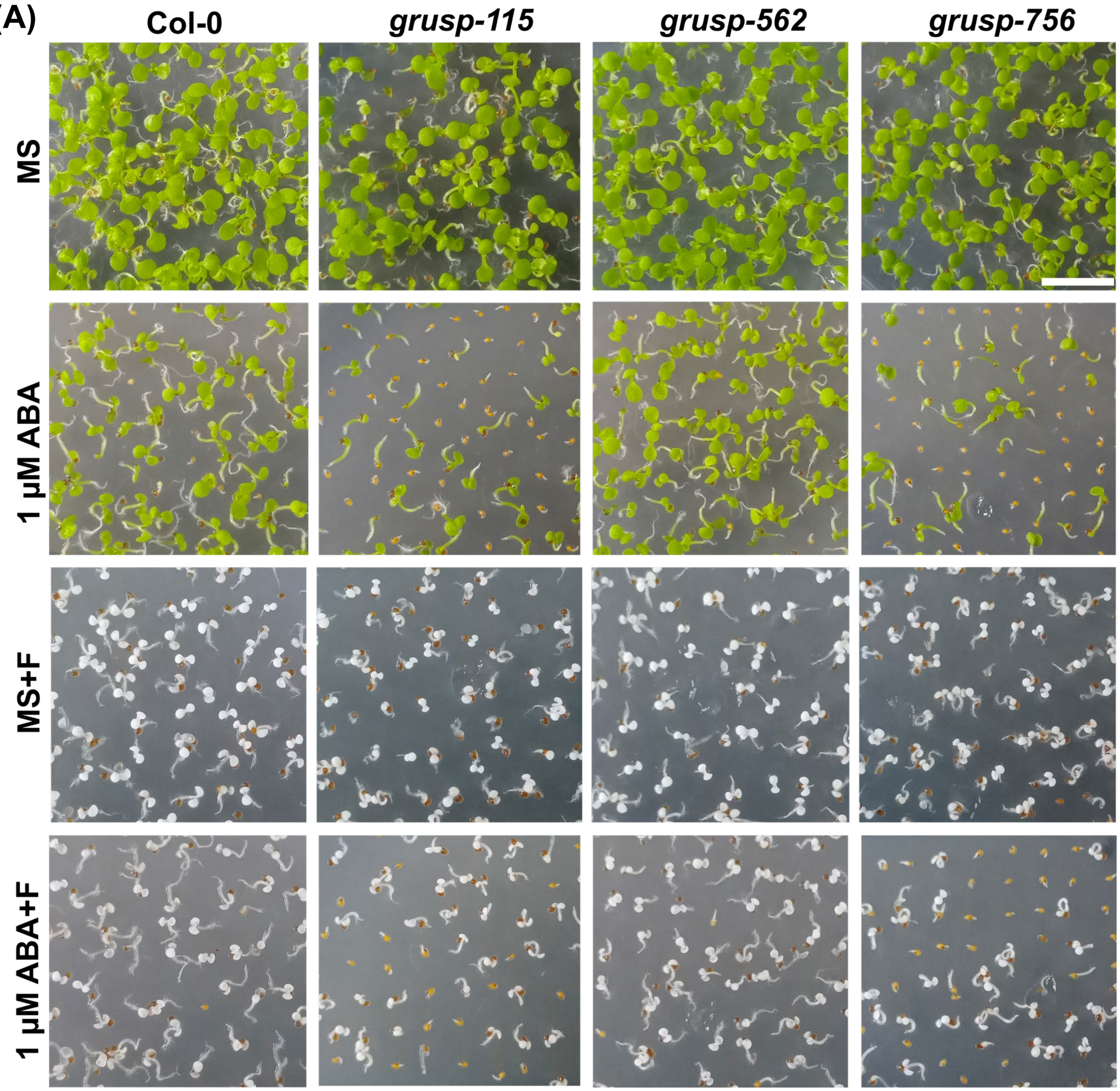

(B)

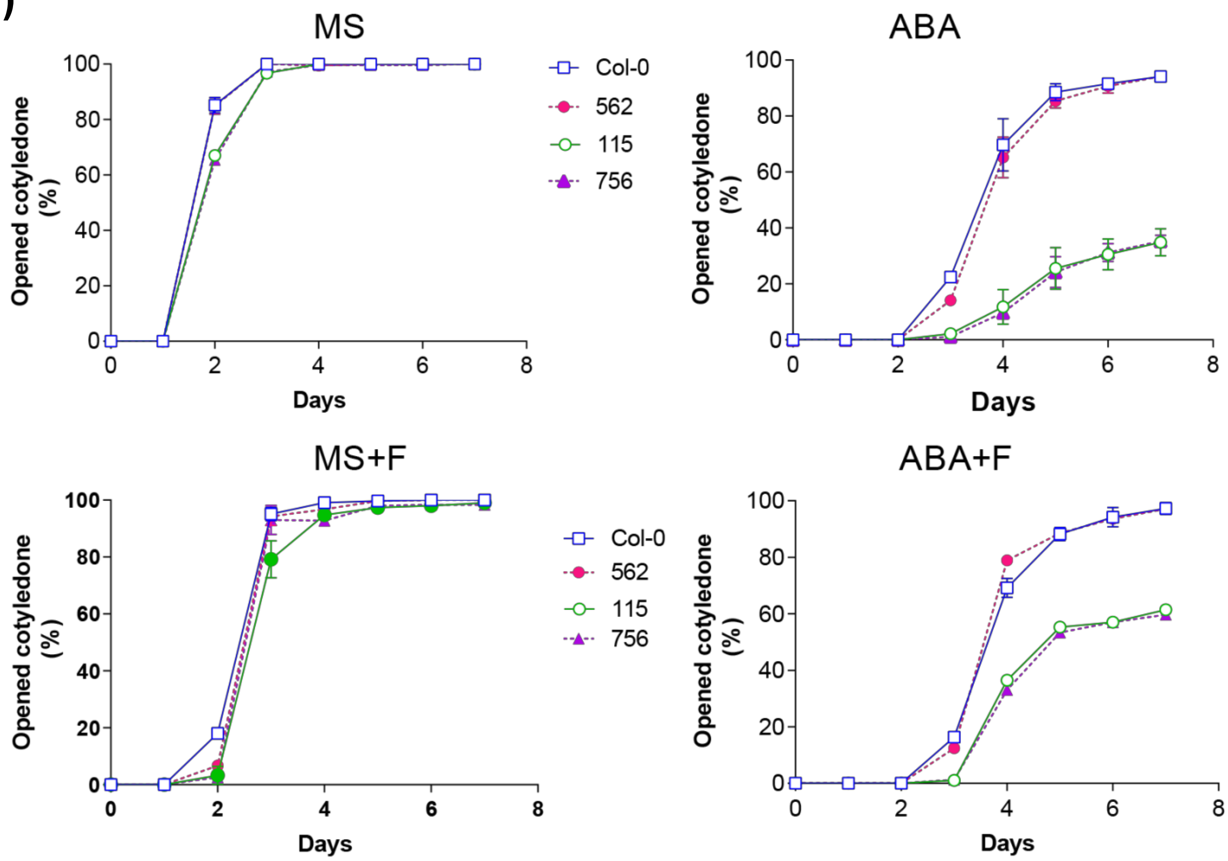

Figure S7. Analysis of ABA sensitivity during seed germination of *grusp-562* and *grusp-756* with suppressed expression of *GRUSP* gene compared with Col-0 and *grusp-115*. (**A**) Photographs of 7-day-old Col-0 and seedlings of *grusp* T-DNA alleles germinated on solid half-strength Murashige and Skoog medium without additions (MS) or supplemented with 1 µM abscisic acid (ABA). For inhibition assays, the seeds were pretreated with 100 µM fluridone (F) for 24 h at 4 °C and then planted on corresponding plates (MS+F or ABA+F). Scale: 2 cm.

(**B**) Percentages of opened cotyledons in 7-day-old plants under normal conditions (MS), in the presence of 1 µM ABA (ABA), with 100 µM fluridone pretreatment without ABA (MS+F), or with fluridone pretreatment in the presence of 1 µM ABA (ABA+F). Each measurement was performed in three biological replicates with 100 seeds per dish.

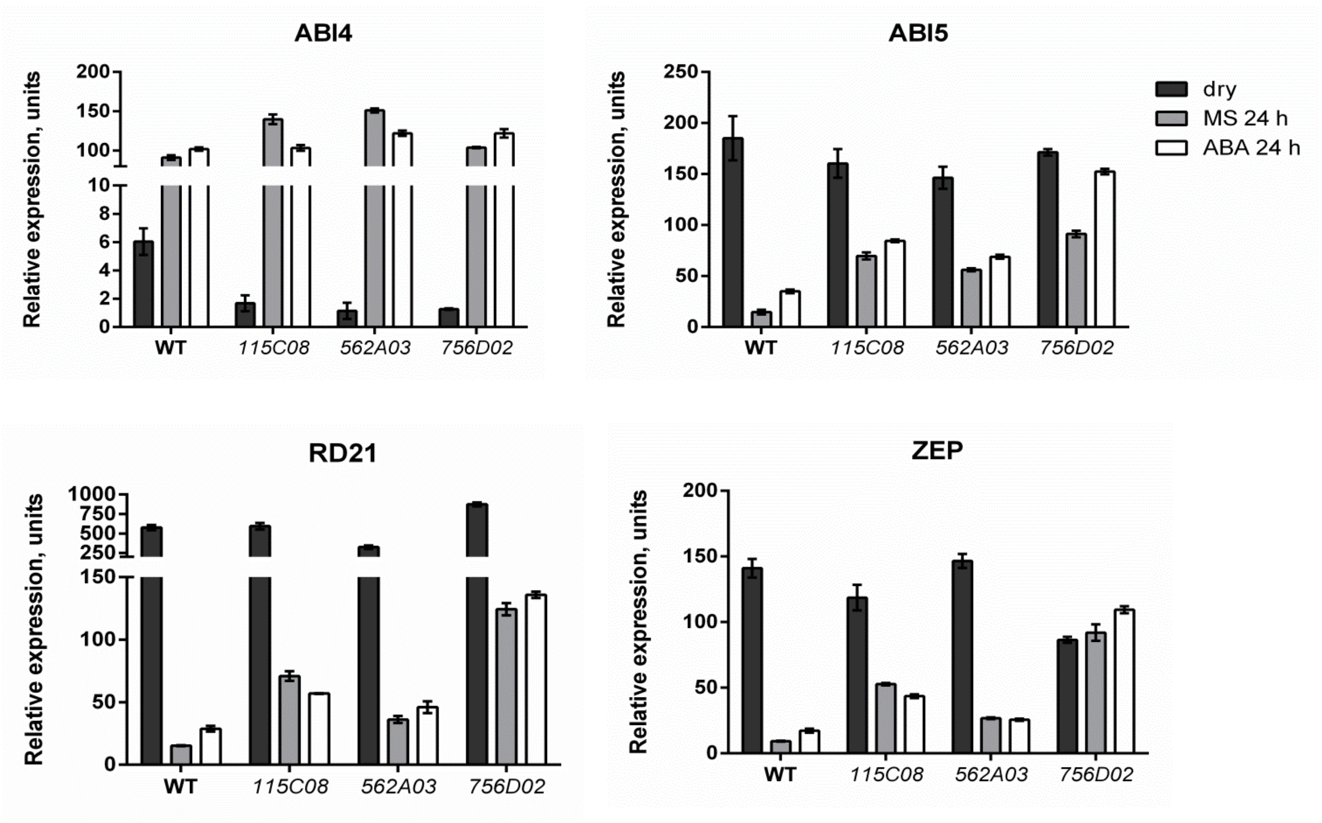

Figure S8. Expression patterns of ABA-responsive genes in Col-0 and *grusp*-mutant seeds. The relative expression levels of genes were analyzed using qRT–PCR in dry seeds (DS) and seeds incubated for 24 h on Murashige and Skoog (MS) medium or with a 50 µM ABA solution under a 16 h light/8 h dark photoperiod at 23 °C with 100 µmol photon m^{-2} s^{-1}. Relative units were normalized to the reference gene *UBC10*. Data represent the means ± SE (*n*=3). Designations: WT, wild type (Col-0); 115C08, *grusp-115;* 562A03, *grusp-562;* 756D02, *grusp-756.*

P1051

caagatacacggaaaagtaacgatcattcttagctcctttgagaaaagaaagaagacgcctgaacaaaagcaaagtctcac
atctaagaatcaaattccacaaaaaaaaagaaaaggatttctgtaatcgtttctcgctttaccaattgcagccggcgaccgtga
agacgggagagtagcactgatctttcaaagatgagaacttttccaaaacccaagtaaatttatcttgcatcttcaaatctaatgac
atgatcacaagttatcgccagtaaaaccctaattcgtaaacaagtaacgtgctttgaaatccagtgtggtagggtttgtgctggtta
tataaacttataagaaaggaaaaataataagctttccatatttcacagacccgagataaaccaaaccatacctgatccaaaat
aaaaattgattcaaactaaaccgaactaggttatttccaaaataagtttgttacggtaattgaaccaaacttgttgttatccgaaact
ABI4
ttgatagaccgaccatctagtcaatatatacttaccacaagaggacctgttcttttgaaaccattgtggaaaataagatatcagaa
caccaaatatgacttagaacactcagcaaacaagatcattgaaagacatagagattctagaggctgacaactctgccttctca
aaacttgatttaagctaattcaaaccgaatttaaatcggttcgttttgggtttatctttttctgagcagaacgaaaccgtaaaattgaa
ABI5
ccaaactcgattctaaaccaaaagccttttctggatattcaaaataaacagttgtctatgttgaaagaaagacacgtgtctatgac
ABI5
acaggtgacgtgtcaatccagtatccatcttaaacatctgttaacactcttcatcttccacctactcaatcacacactagcattacgt
aacgtatggtacgatctaaattatatacatatcaaaaagtggctggagccgaacaaaaaaaacatacgatagagaaaacgt
acggagtaaaaaatggagacttacgttg*ATG*
5’-UTR

Figure S9. The nucleotide sequence of the promoter region of the *GRUSP* gene, 1000 bp in length, including the 5’-UTR region (underlined). The basal promoter sequence of the gene, −1051 bp from the ATG (in red) initiation codon, was identified using the PlantProm DB program (http://www.softberry.com/, accessed 14 July 2026). Position of ABI4- and AB5-sensitive *cis*-regulatory elements identified by PlantPAN5.0 database (https://plantpan.itps.ncku.edu.tw/plantpan5/, accessed 14 July 2026).

Adenine nucleotide alpha hydrolases-like superfamily protein [Arabidopsis thaliana]

NCBI Reference Sequence: NP_850717.1

GenPept Identical Proteins Graphics

>NP_850717.1 Adenine nucleotide alpha hydrolases-like superfamily protein [Arabidopsis thaliana]

M**E**TYVDAIGEDTAATTTTAETAANKNKKKLKVMVAIDESKNSFDALEWAVDHLRVVISAEPETGQEGGLL
TLLHVHPTYLQYIYPSGGTDSVPEPMRKAREESTTNLFTRALEICRGKMVKTETMILEGDPKEMICQAVE
QTHVDLLVVGSRGLGMIKRAFLGSVSDYCAQHAKCPILIVRPPRETSTSNTKEHKSK

NCBI Reference Sequence: NP_191404.2

GenPept Identical Proteins Graphics

>NP_191404.2 Adenine nucleotide alpha hydrolases-like superfamily protein [Arabidopsis thaliana]

M**E**TYVDAIGEDTAATTTTAETAANKNKKKLKVMVAIDESKNSFDALEWAVDHLRVVISAEPETGQEGGLL
TLLHVHPTYLQYIYPSGGTASAVYATDSVPEPMRKAREESTTNLFTRALEICRGKMVKTETMILEGDPKE
MICQAVEQTHVDLLVVGSRGLGMIKRAFLGSVSDYCAQHAKCPILIVRPPRETSTSNTKEHKSK

Figure S10. Amino acid sequences of two GRUSP forms revealed the presence of an N-terminal methionine followed by a destabilizing glutamate (E), recognized by the Arg/N-end rule pathway, which is associated with rapid protein turnover. This suggests that GRUSP is likely a short-lived protein.
